# miR-34a and IRE1A/XBP-1(S) Form a Double-Negative Feedback Loop to Regulate Hypoxia-Induced EMT, Metastasis, Chemo-Resistance and Autophagy

**DOI:** 10.3390/cancers15041143

**Published:** 2023-02-10

**Authors:** Nassim Bouznad, Matjaz Rokavec, Meryem Gülfem Öner, Heiko Hermeking

**Affiliations:** 1 Experimental and Molecular Pathology, Institute of Pathology, Ludwig-Maximilians-Universität, Thalkirchner Strasse 36, 80337 Munich, Germany; 2 German Cancer Consortium (DKTK), Partner Site Munich, 80336 Munich, Germany; 3 German Cancer Research Center (DKFZ), 69120 Heidelberg, Germany

**Keywords:** p53, miR-34a, miR-34, IRE1A, XBP-1(S), *XBP-1*, HIF1A, colorectal cancer, ER stress, unfolded protein response, autophagy, hypoxia

## Abstract

**Simple Summary:**

The hypoxic tumor microenvironment is a key factor in the formation of metastasis and treatment resistance. We recently identified a new regulatory network consisting of the hypoxia-inducible transcription factor HIF-1A and the p53-inducible miR-34a that determines whether a tumor cell undergoes EMT (epithelial-mesenchymal transition) or MET (mesenchymal-epithelial transition) under hypoxic conditions. Here, we characterized XBP-1 and IRE1A as new miR-34a targets, which are relevant in this context. In addition, we found that the activation of the IRE1A/XBP-1 arm of the unfolded protein response (UPR) by hypoxia results in repression of *miR-34a* and thereby mediates hypoxia-induced EMT, migration, invasion, and chemo-resistance in *p53* mutated/deficient CRC lines and ultimately contributes to lung metastasis formation. In this context, the restoration of miR-34a may be of therapeutic value in the future.

**Abstract:**

Tumor-associated hypoxia, i.e., decreased availability of oxygen, results in a poor clinical outcome since it promotes EMT, metastasis, and chemotherapy-resistance. We have previously identified p53 and its target miR-34a, as critical determinants of the effect of hypoxia on colorectal cancer (CRC). Here, we aimed to characterize mechanisms that contribute to the selective advantage of cells with loss of p53/miR-34a function in a hypoxic environment. Using in silico prediction, we identified XBP-1 and IRE1A as potential miR-34a targets. IRE1A and XBP-1 are central components of the unfolded protein response that is activated by ER stress, which is also induced in tumor cells as a response to harsh conditions surrounding tumors such as hypoxia and a limited supply of nutrients. Here we characterized the XBP-1(S) transcription factor and its regulator IRE1A as direct, conserved miR-34a targets in CRC cells. After hypoxia and DNA damage, IRE1A and XBP-1 were repressed by p53 in a miR-34a-dependent manner, whereas *p53*-deficient cells showed induction of IRE1A and XBP-1(S). Furthermore, miR-34a expression was directly suppressed by XBP-1(S). In *p53*-deficient CRC cells, hypoxia-induced EMT, migration, invasion, metastases formation, and resistance to 5-FU were dependent on IRE1A/XBP-1(S) activation. Hypoxia-induced autophagy was identified as an XBP-1(S)-dependent mediator of 5-FU resistance and was reversed by ectopic miR-34a expression. The HIF1A/IRE1A/XBP-1(S)/p53/miR-34a feedback loop described here represents a central regulator of the response to hypoxia and ER stress that maintains cellular homeostasis. In tumors, the inactivation of *p53* and *miR-34a* may result in IRE1A/XPB-1(S)-mediated EMT and autophagy, which ultimately promotes metastasis and chemoresistance.

## 1. Introduction

The hypoxic microenvironment found in tumors is one of the major drivers of the metastatic spread of cancer, however, the underlying mechanisms are not completely understood [1]. Intra-tumoral hypoxia is associated with metastasis, therapy resistance, and poor clinical outcome in CRC [2,3]. Hypoxia promotes metastasis in CRC by inducing EMT (epithelial-mesenchymal transition) [4,5], a developmental program that is commonly activated in tumor cells, which facilitates invasion and migration toward blood vessels [6]. Hypoxia leads to the activation of the transcription factor HIF-1A, which promotes EMT/epithelial-mesenchymal transition and metastasis [7]. The p53 tumor suppressor protein is also activated by hypoxia [8], which is, at least in part, due to the direct interaction of p53 with HIF-1A [9]. The interplay between HIF-1A and p53 may therefore serve as a critical determinant of cancer invasion and metastasis under hypoxic conditions. Recently, we demonstrated that the p53 status and the resulting alterations in the expression of miR-34a critically influence the decision as to whether colorectal cancer cells undergo EMT or MET under hypoxic conditions [10]. We demonstrated that in *p53*-deficient colorectal cancer cell lines, direct repression of *miR-34a* by HIF-1A is necessary for hypoxia-induced EMT, invasion, and migration, whereas, in cells with wild-type p53, miR-34a expression was induced by hypoxia-activated p53 and mediated MET. In addition, we determined that *PPP1R11/INH3* is directly inhibited by miR-34a in this context, resulting in the inactivation of STAT3 by protein phosphatase (PP1) mediated dephosphorylation [10].

Hypoxia activates a range of cellular stress-response pathways, including the unfolded protein response (UPR), which is activated when improper folding and maturation of secretory-pathway proteins occurs in the endoplasmic reticulum (ER) resulting in so-called ER stress [11,12]. Sustained activation of ER stress sensors, such as IRE1A, PERK, and ATF6, enhances tumorigenesis, metastasis, and drug resistance [13,14]. IRE1A is essential for cell viability under stress conditions that cause unfolded proteins to accumulate in the ER [15]. IRE1A is a type I ER-resident transmembrane protein with an ER luminal dimerization domain and a cytoplasmic domain with Ser/Thr kinase and endoribonuclease activities [16]. The latter is involved in the alternative splicing of *XBP-1* mRNA resulting in *XBP-1(S)*, the spliced version, and *XBP-1(U)*, the un-spliced isoform. XBP-1(S) encodes a basic-region leucine zipper (bZIP) transcription factor localized in the nucleus, whereas XBP-1(U) resides in the cytoplasm [17]. The UPR regulates the balance between survival and apoptosis, as well as between dormancy and aggressive growth of tumor cells [18,19]. Importantly, the UPR is required for tumor cell proliferation under hypoxic conditions, as it protects tumor cells against hypoxia-induced cell death [20]. XBP-1(S) is a critical transcriptional activator of the UPR and functions as an essential cofactor of the HIF-1A transcriptional complex [21]. Xi Chen and colleagues found an enrichment of the HIF-1A motif in XBP-1 Chip-Seq results, and suggested that XBP-1 and HIF1A might interact within the same transcriptional complex [21]. They revealed a physical interaction of HIF-1A with XBP-1(S) in cells co-expressing HIF-1A and XBP-1 cultured under hypoxic conditions. Moreover, this interaction occurs in the nucleus, and XBP-1 and HIF-1A co-occupied several well-known HIF-1A targets [21]. XBP-1 co-localizes with hypoxia markers in tumors and the loss of *XBP-1* increases the sensitivity of tumor cells to hypoxia-induced apoptosis and inhibits tumor growth, implicating XBP-1(S) as a critical survival factor under hypoxia [22]. The pro-survival effect of XBP-1 has also been linked to the induction of autophagy [23].

In order to identify additional miR-34a-regulated effectors of hypoxia similar to *PPP1R11/INH3* [10], we performed a bioinformatics screen and identified XBP-1 and IRE1A as potential candidates. We characterized XBP-1(S) and IRE1A as direct miR-34a targets. Furthermore, hypoxia resulted in the IRE1A-mediated activation of XBP-1(S), which directly repressed *miR-34a* in *p53*-deficient tumor cells. Therefore, IRE1A, XBP-1(S), and miR-34a form a double negative regulatory loop, which selectively stimulates EMT, migration, invasion, and metastasis in *p53*-deficient cells.

## 2. Materials and Methods

### 2.1. Cell Culture

DLD-1, HCT-15, HT29, CT26, HCT116, and RKO cell lines were maintained in McCoy’s 5A Medium, SW480 and SW620 were maintained in Dulbecco’s Modified Eagle’s Medium (Invitrogen, Carlsbad, CA, USA). *TP53^+/+^* and *TP53^−/−^* HCT116 and RKO lines were kindly provided by Bert Vogelstein (Johns Hopkins University, Baltimore, MD, USA) and CT26 cells by Gabriele Multhoff (Technical University, Munich, Germany). All cells were cultured in medium supplemented with 10% fetal bovine serum (FBS, Invitrogen) and in the presence of 100 U/mL penicillin and 0.1 mg/mL streptomycin at 20% O_2_ and 5% CO_2_ at 37 °C. Hypoxia was achieved using a CD210 incubator (Binder, Tuttlingen, Germany). Doxycycline (DOX; Sigma, St Louis, MO, USA) was used at a final concentration of 100 ng/mL. STF083010 (SML0409, Sigma-Aldrich) was used at a concentration of 60 µM, SBI-0206965 (SML1540, Sigma-Aldrich) was used at a concentration of 10 µM, chloroquine (Sigma) was used at a concentration of 20 µM, Etoposide (Sigma-Aldrich) was used at a concentration of 20 mM and 5-fluorouracil (5-FU) (Sigma-Aldrich) at 25 mg/mL. Small interfering RNAs (Ambion silencer siRNAs, Foster City, CA, USA): negative control [ID 4611], *XBP-1* ID [s14914], *IRE1A* ID [s200432], *HIF1A* ID [s6530]. A FlexiTube Gene Solution was used to knock down *XBP-1* (GS7494, 1027416; Qiagen, Hilden, Germany) and contains 4 different oligos: SI04352551, SI04224899, SI04143188, SI03226895. siRNAs were transfected at a final concentration of 10 nM using Lipofectamin (Qiagen).

### 2.2. RNA Isolation and qPCR

Total RNA was isolated from human cell lines with the Total RNA Isolation Kit (Roche, Basel, Switzerland) and from mouse tissues with the RNeasy Total RNA Isolation Kit (Qiagen) according to manufacturer’s instructions. 1μg of total RNA per sample was used to generate cDNA using the Verso cDNA synthesis kit (Thermo Scientific, Waltham, MA, USA). Quantitative real-time PCR (qPCR) was performed with the Fast SYBR Green Master Mix (Applied Biosystems, Waltham, MA, USA) on a LightCycler 480 (Roche). The sequences of oligonucleotides used as qPCR primers are listed in Supplementary Table S1. Expression of mRNA and primary miRNA was normalized to GAPDH. The qPCR results were calculated using the ΔΔCt method. Experiments were performed in triplicate.

### 2.3. Western Blot Analysis

Western blot analyses were performed as described previously. In brief, cells were lysed in RIPA lysis buffer with Complete Mini Protease and Phosphatase Inhibitors (Roche) added, sonicated, and centrifuged at 16.060× *g* for 15 min at 4 °C. For mouse tissues, samples were lysed in ice-cold lysis buffer by mechanical homogenization as described previously [24]. To complete lysis, samples were incubated 10 min on ice and centrifuged at 13,000 rpm at 4 °C to separate cell debris and the lysate for 15 min. Protein concentration was measured with BCA Protein Assay Kit (Thermo Fisher Scientific) according to manufacturer’s instructions. 30–60 μg of whole cell lysate per lane was separated on SDS-acrylamide gels and transferred into Immobilon PVDF membranes (Millipore, Burlington, MA, USA). For immunodetection, membranes were incubated with antibodies listed in Supplementary Table S2. Enhanced chemiluminescence (ECL, Millipore) signals were recorded with a 440CF imaging system (Eastman Kodak Co., Rochester, NY, USA). For quantification of Western blot signals, intensities of protein expression signals were quantified by densitometric analysis. The resulting values of the protein of interest were normalized to the corresponding loading controls.

### 2.4. Indirect Immunofluorescence and Confocal Laser-Scanning Microscopy

Cells cultivated on glass cover-slides were fixed in 4% paraformaldehyde/PBS for 10 min, permeabelized in 0.2% Triton × 100 (Sigma-Aldrich, St. Louis, MO, USA) for 20 min, and blocked in 100% FBS for 30 min. Antibodies are listed in Supplementary Table S2. Chromatin was stained by DAPI (Carl Roth, Karlsruhe, Germany). Slides were covered with ProLong Gold Antifade (Invitrogen, Waltham, MA, USA). CLSM (confocal laser scanning microscopy) images were captured with an LSM700 microscope using a Plan Apochromat 20×/0.8 M27 objective and ZEN 2009 software (Zeiss, Jena, Germany).

### 2.5. Modified Boyden-Chamber Assay for Analysis of Migration and Invasion

Migration and invasion analyses were conducted as described previously. In short, cells were serum-starved by cultivation in 0.1% serum for 24 h. To analyze migration, 5 × 10^4^ cells were seeded in the upper chamber (8.0 μm pore size membrane; Corning, Corning, NY, USA) in serum-free medium. To analyze invasion, chamber membranes were first coated with Matrigel (BD Bioscience, Franklin Lakes, NJ, USA) at a dilution of 3.3 ng/mL in medium without serum. Then 8 × 10^4^ cells were seeded on the Matrigel in the upper chamber in serum-free medium. 10% FBS was placed as a chemo-attractant in the lower chamber. After cells were cultured for 48 h, non-motile cells at the top of the filter were removed and the cells in the bottom chamber were fixed with methanol, stained with DAPI, and counted using immunofluorescence microscopy. Relative invasion/migration was normalized to the corresponding control.

### 2.6. Chromatin Immunoprecipitation (ChIP) Assay

Chromatin immunoprecipitation in DLD-1 cells was performed according to instructions provided in iDeal ChIP-qPCR kit (Diagenode, Ougrée, Belgium). The sequences of oligonucleotides used as qChIP primers are listed in Supplementary Table S3.

### 2.7. Dual 3′-UTR Luciferase Reporter Assays

The full-length 3′-UTRs of the human and mouse *XBP-1* and *IRE1A* mRNA were PCR-amplified from cDNA of human diploid fibroblasts (HDFs). The PCR product was cloned into the shuttle vector pGEM-T-Easy (Promega, Madison, WI, USA), and then transferred into the pGL3-control-MCS vector and verified by sequencing. Mutagenesis of the *miR-34a* seed-matching sequences in human and mouse was achieved with the Quik-Change II XL Site-Directed Mutagenesis Kit (Stratagene, San Diego, CA, USA) according to manufacturer’s instructions and verified by sequencing. H1299 cells were seeded in 12-well format dishes at 3 × 10^4^ cells/well for 24 h, transfected 100 ng of the respective firefly luciferase reporter plasmid, 20 ng of *Renilla* reporter plasmid as a normalization control, and 25 nM of *pre-miR-34a* (Ambion, PM11030,) or a negative control oligonucleotide (Ambion, neg. control #1) with HiPerFect Transfection Reagent (Qiagen) for 48 h. The analysis was performed with Dual Luciferase Reporter assay (Promega) according to manufacturer’s instructions. Luminescence intensities were measured with an Orion II luminometer (Berthold, Berthold, ND, USA) in 96-well format and analyzed with the SIMPLICITY software package (DLR) version 3.0. The sequences of oligonucleotides used as primers are listed in Supplementary Table S4.

### 2.8. Wound Healing Assay

The wound healing assay was performed as described previously. In brief, Mitomycin C [10 ng/mL] was added two hours before generating a scratch using a Culture-Insert (IBIDI, 80241). After washing twice with HBSS to remove Mitomycin C and detached cells, medium was added. Cells were allowed to close the wound for the indicated periods. Images were captured on an Axiovert Observer Z.1 microscope connected to an AxioCam MRm camera using the Axiovision software (Zeiss) at the respective time-points.

### 2.9. Animal Experiments

The generation of *miR-34a*^−/−^ mice with a C57BL6/SV129 background has been described previously [25]. To delete *Mir34a* and *p53* in IECs, *Mir34a*^Fl/Fl^ [25] and p53^Fl/Fl^ [24] mice crossed to *Villin-Cre* mice [26]. *p53^Fl/Fl^* mice were obtained from Anton Berns (NKI, Amsterdam, The Netherlands) and *Villin-Cre* mice from Jackson Laboratories (Bar Harbor, ME, USA). All mice were crossed to at least 5 generations to FVB background. In all experiments, littermate controls were used. Mice were injected i.p. with Azoxymethane (AOM, 10 mg/kg, Sigma-Aldrich) at the age of 6 to 10 weeks, six times in weekly intervals, and on week 16 all mice were sacrificed. Mice were housed in individually ventilated cages (IVC) using “Lingocel Select” bedding. All animal protocols were approved by the local authorities (Regierung von Oberbayern, AZ: 55.2-1-54-2532-201-2014). All experiments involving mice were conducted with approval by the local Animal Experimentation Committee (Regierung of Oberbayern).

### 2.10. Analysis of Metastases Formation in NOD/SCID Mice

NOD/SCID mice were purchased from the Jackson Laboratory. DLD-1-luc2 cells transfected with *XBP-1* or control siRNAs, or treated with DMSO or STF-083010 (50 µM) for 24 h and cultured at 20% O_2_ or 0.5% O_2_ for 30 h. 4 × 10^6^ DLD-1-luc2 cells were dissolved in 0.2 mL HBSS and were injected into the lateral tail vein of 6–8 week old male immuno-compromised NOD/SCID mice using 25-gauge needles. Anesthetized mice were injected intraperitoneally with D-luciferin (150 mg/kg) and imaged 10 min after injection using the IVIS Illumina System (Caliper Life Sciences, Hopkinton, MA, USA) in weekly intervals during light cycles. The acquisition time was 2 min. After 9 weeks mice were sacrificed and resected lungs examined for metastases using H&E staining. All experiments involving mice were conducted with approval by the local Animal Experimentation Committee (ROB-55.2-2532.Vet_02-18-57, Regierung of Oberbayern). All experiments were performed in accordance with the ARRIVE guidelines and regulations.

### 2.11. Tissue Preparation and Immunohistochemistry

The colon was opened longitudinally and rolled to form a “swiss roll”. Tissues were fixed overnight in formalin, dehydrated, and embedded in paraffin and 2 μm sections were obtained. For immunohistochemistry (IHC) staining, tissue sections were deparaffinized/rehydrated, then boiled in citrate-based antigen retrieval solution (S2369, Dako, Hamburg, Germany) for 20 min, incubated in 3% H_2_O_2_/PBS (Carl Roth, 9681.1) for 10 min, and then blocked with 2.5% Normal Horse Serum (Vector Labs, San Francisco, CA, USA). The serial sections were incubated overnight at 4 °C with the primary antibody (anti-XBP-1(S), Cell Signaling #12782), followed by detection reagent (ImmPRESS^®^ HRP Horse Anti-Rabbit IgG Polymer Detection Kit, Peroxidase, MP-7401) for 1 h at room temperature. Bound antibodies were visualized by using DAB staining (Dako Liquid + Dab Substrate Chromogen System, K3468, Dako) according to the manufacturer’s instructions. Sections were then counter-stained with hematoxylin (H-3401 Vector Labs). Slides were scanned with Vectra^®^ Polaris™ Automated Quantitative Pathology Imaging System (PerkinElmer, Waltham, MA, USA) and quantified by Image J software (U. S. National Institutes of Health, Bethesda, MD, USA).

### 2.12. Statistical Analysis

The GraphPad Prism 8.3.0 software was used for statistical analyses. The statistical significance of differences between group means was determined with the two-tailed unpaired Student’s *t*-test and one-way ANOVA. Kaplan–Meier curves were used to display the overall survival time and the results were compared with a log-rank test. *p* values less than 0.05 were considered as statistically significant with asterisks indicated (* *p* < 0.05, ** *p* < 0.01, *** *p* <0.001, or **** *p* < 0.0001).

## 3. Results

### 3.1. XBP-1 Is a Direct Target for Repression by miR-34a

**Figure 1:**
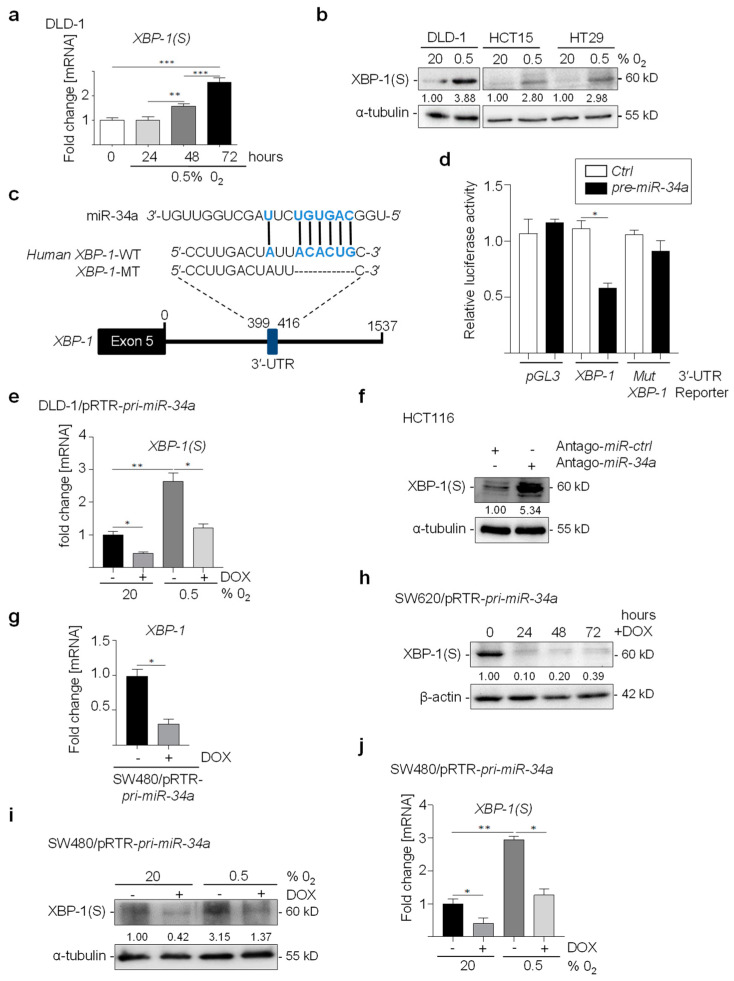
Hypoxia-induced *XBP-1* is repressed by miR-34a. (**a**) qPCR analysis of *XBP-1(S)* in DLD-1 cells exposed to 20% or 0.5% O_2_ for the indicated times. (**b**) Western blot analysis of XBP-1(S) in DLD-1, HCT15, and HT-29 cells exposed to 20% or 0.5% O_2_ for 72 h. (**c**) Schematic representation of the *XBP-1* 3′-UTRs indicating the miR-34a seed-matching sequences (SMS) and its mutagenesis. The vertical bars indicate possible base pairing. (**d**) Dual reporter assay after transfection of H1299 cells with *pre-miR-34a* oligonucleotides and human *XBP-1* 3′-UTR reporter constructs. (**e**) qPCR analysis of *XBP-1(S)* in DLD-1 cells harboring a pRTR/*pri-miR-34a* vector after addition of DOX and exposed to 20% or 0.5% O_2_ for 72 h. (**f**) Western blot analysis of XBP-1(S) in HCT116 cells transfected with antago-miR-34a or antago-miR negative control oligonucleotides. (**g**) qPCR analysis of *XBP-1* in SW480 cells harboring a pRTR/*pri-miR-34a* vector after addition of DOX for 48 h. (**h**) Western blot analysis of XBP-1(S) and XBP-1(U) in SW620 cells harboring a pRTR/pri-miR-34a vector after addition of DOX. (**i**,**j**) Western blot and qPCR analysis of XBP-1(S) in SW480 cells harboring a pRTR/*pri-miR-34a* vector after addition of DOX and exposure to 20% or 0.5% O_2_ for 72 h. In panel (**a**,**d**,**e**,**h**,**j**) mean values ± SD (n = 3) are provided. (***) *p* < 0.001, (**) *p* < 0.01, (*) *p* < 0.05.

When the CRC cell line DLD-1 was exposed to hypoxia for up to three days, a significant induction of XBP-1(S) at the mRNA and protein levels was observed (Figure 1a,b). In HCT-15 and HT-29 CRC cells, XBP-1(S) protein was also induced by hypoxia (Figure 1b). We have previously shown that miR-34a is directly repressed by HIF1A in CRC cell lines [10]. As a consequence, miR-34a targets are induced by hypoxia, such as *INH3* [10]. Therefore, we asked whether XBP-1 contains a miR-34a seed matching site (SMS). Inspection of the human *XBP-1* 3′-UTRs using the TargetSCAN algorithm revealed a miR-34 seed-matching sequence (SMS), suggesting that *XBP-1* may represent a miR-34a target (Figure 1c). Indeed, a human *XBP-1* 3′-UTR reporter was repressed after ectopic expression of *pre-miR-34a*, whereas a reporter with a mutant miR-34a SMS was refractory (Figure 1d). Furthermore, the induction of *XBP-1(S)* by hypoxia was prevented by ectopic *pri-miR-34a* expression in DLD-1 (Figure 1e). In addition, XBP-1(S) protein levels increased after transfection of miR-34a-specific antagomir in HCT116 cells (Figure 1f). Moreover, ectopic expression of *pri-miR-34a* in SW480 cells resulted in the repression of endogenous *XBP-1* mRNA expression (Figure 1g). In line with these results, ectopic *pri-miR-34a* expression repressed XBP-1 (S) protein in a time-dependent manner in SW620 cells (Figure 1h). In addition, the induction of XBP-1(S) by hypoxia was prevented by ectopic *pri-miR-34a* expression in SW480 cells at the mRNA and protein levels (Figure 1i,j) at the mRNA and protein levels. Taken together, these results show that *XBP-1* is directly repressed by miR-34a and that this repression is alleviated during the response to hypoxia.

### 3.2. IRE1A Is Directly Repressed by miR-34a

**Figure 2:**
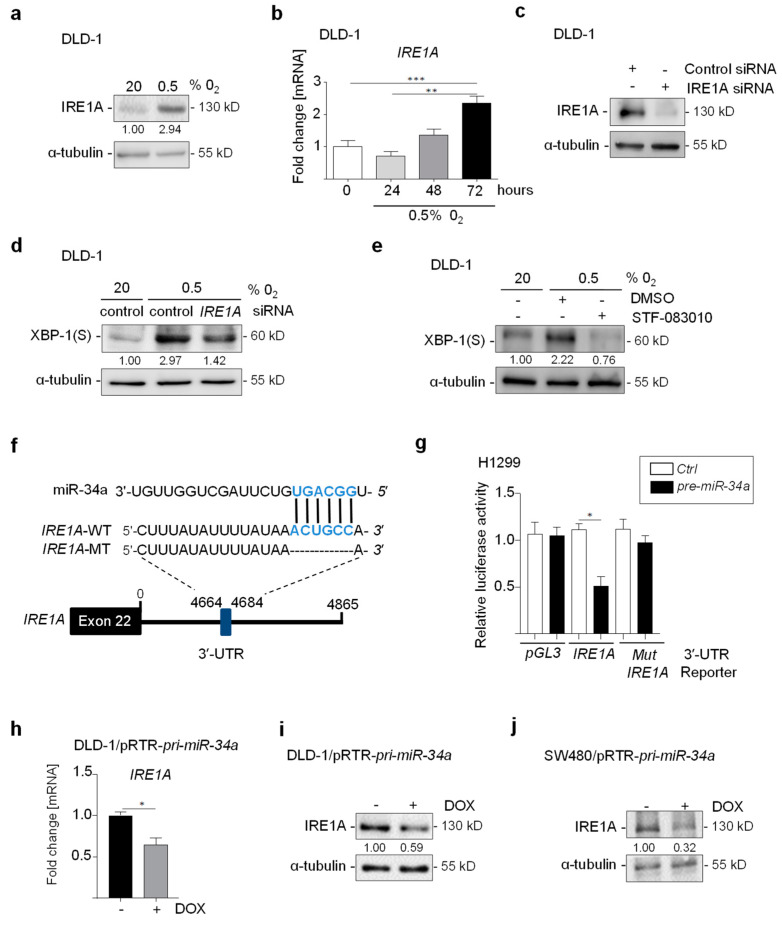
Hypoxia-induced *IRE1A* is repressed by miR-34a. (**a**) Western blot analysis of IRE1A in DLD-1 cells exposed to 20% or 0.5% O_2_ for 48 h. (**b**) qPCR analysis of *IRE1A* expression in DLD-1 cells exposed to 20% or 0.5% O_2_ for the indicated times. (**c**) Western Blot analysis of IRE1A in DLD-1 cells transfected with *IRE1A* or control siRNAs. (**d**) Western Blot analysis of XBP-1(S) in DLD-1 cells transfected with *IRE1A* or control siRNAs for 24 h, then cultured at 20% or 0.5% O_2_ for 72 h. (**e**) Western blot analysis of XBP-1(S) in DLD-1 cells treated with DMSO or STF-083010 (60 µM) for 24 h, then cultured at 20% O_2_ or 0.5% O_2_ for 72 h. (**f**) Schematic representation of the *IRE1A* 3′-UTR indicating the miR-34a seed-matching sequences (SMS) and mutagenesis. The black vertical bars indicate possible base pairing. (**g**) Dual reporter assay after transfection of H1299 cells with *pre-miR-34a* oligonucleotides and human *IRE1A* 3′-UTR reporter constructs. (**h**–**j**) qPCR and Western blot analysis of IRE1A in DLD-1 and SW480 cells harboring a pRTR/pri-miR-34a vector after addition of DOX for 48 h. In panel (**b**,**g**,**h)**, mean values ± SD (n = 3) are provided. (***) *p* < 0.001, (**) *p* < 0.01, (*) *p* < 0.05.

Hypoxia-induced IRE1A mRNA and protein expression in DLD-1 (Figure 2a,b). *XBP-1* mRNA is spliced by IRE1A in response to hypoxia-induced ER stress to produce *XBP-1(S)*, which encodes a transcription factor [17]. Indeed, siRNA-mediated knockdown of *IRE1A* (Figure 2c), prevented the induction of XBP-1(S) by hypoxia (Figure 2d). Moreover, treatment of DLD-1 cells with STF-083010, an inhibitor of the endonuclease activity of IRE1A, prevented the induction of XBP-1(S) by hypoxia (Figure 2e). In order to determine whether repression of miR-34a contributes to the induction of IRE1A levels under hypoxic conditions, we used the TargetSCAN algorithm to identify a putative miR-34a SMS in the *IRE1A* 3′-UTR. (Figure 2f). A human *IRE1A* 3′-UTR reporter was repressed after transfection of *pre-miR-34a*, whereas a reporter with a mutant SMS was refractory to *pre-miR-34a* (Figure 2g). Furthermore, ectopic expression of *pri-miR-34a* in DLD-1 and SW480 CRC cells resulted in the repression of IRE1A at the mRNA and the protein levels (Figure 2h–j). Taken together, these results show that *IRE1A* is a direct target of miR-34a.

### 3.3. p53 Represses XBP-1(S) and IRE1A via miR-34a

**Figure 3:**
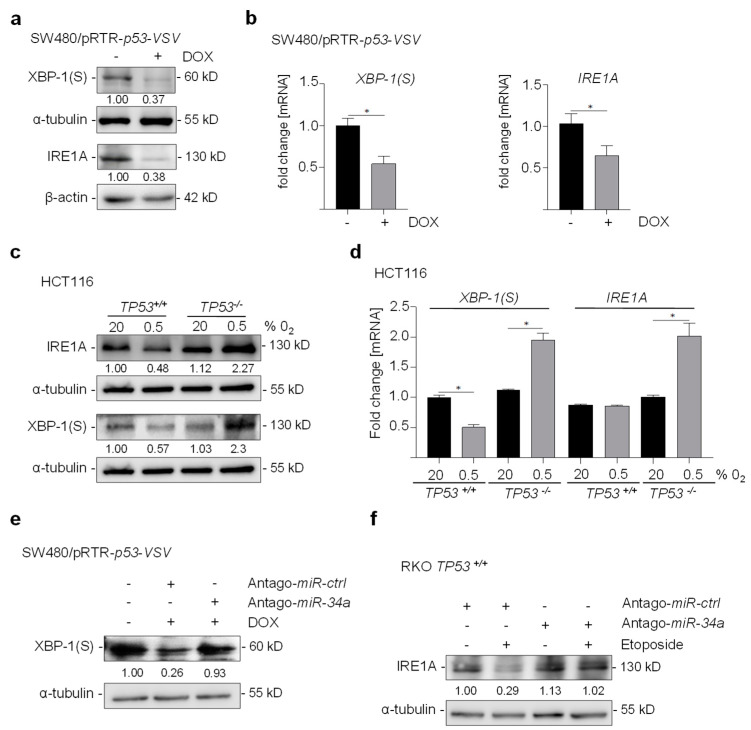
Negative regulation of XBP-1(S) and IRE1A by p53 is mediated by miR-34a. (**a**,**b**) Western blot and qPCR analysis of XBP-1(S) and IRE1A in SW480 cells harboring a pRTR/*p53* vector after addition of DOX for 72h. (**c**,**d**) Western blot and qPCR analysis of XBP-1(S) and IRE1A in HCT116 *TP53^−/−^* and *TP53^+/+^* cells exposed to 20% or 0.5% O_2_ for 48 h. (**e**) Western blot analysis of XBP-1(S) in SW480/pRTR-p53-VSV cells transfected with antago-miR-34a or antago-miR negative control oligonucleotide for 24 h and/or subsequently treated with DOX for 48 h. (**f**) Western blot analysis of IRE1A in RKO *TP53^+/+^* cells transfected with antago-miR-34a or antago-miR-negative control oligonucleotide for 24 h and/or subsequently treated with etoposide (20 mM) for 48 h. In panel (**b**,**d**,**f**) mean values ± SD (n = 3) are provided. (*) *p* < 0.05.

Consistent with their inhibition by miR-34a, XBP-1(S) and IRE1A were repressed by ectopic p53 in SW480 CRC cells at the mRNA and protein levels (Figure 3a,b). Furthermore, hypoxia repressed XBP-1(S) and IRE1A at the protein and mRNA levels in *p53*-proficient HCT116 cells, whereas it resulted in their up-regulation in *p53*-deficient, isogenic HCT116 cells (Figure 3c,d). The repression of XBP-1(S) by p53 was alleviated by the transfection of miR-34a-specific antagomirs (Figure 3e). Therefore, miR-34a mediates the repression of XBP-1(S) and IRE1A by p53. In addition, the repression of IRE1A after DNA damage was prevented by miR-34a-specific antagomirs (Figure 3f). Taken together, the results demonstrate the CRC cells display a differential regulation of IRE1A and XBP-1(S) by miR-34a in response to hypoxia depending on their *p53* status.

### 3.4. Conservation of miR-34a-Mediated Repression of XBP-1 and IRE1A in Mice

**Figure 4:**
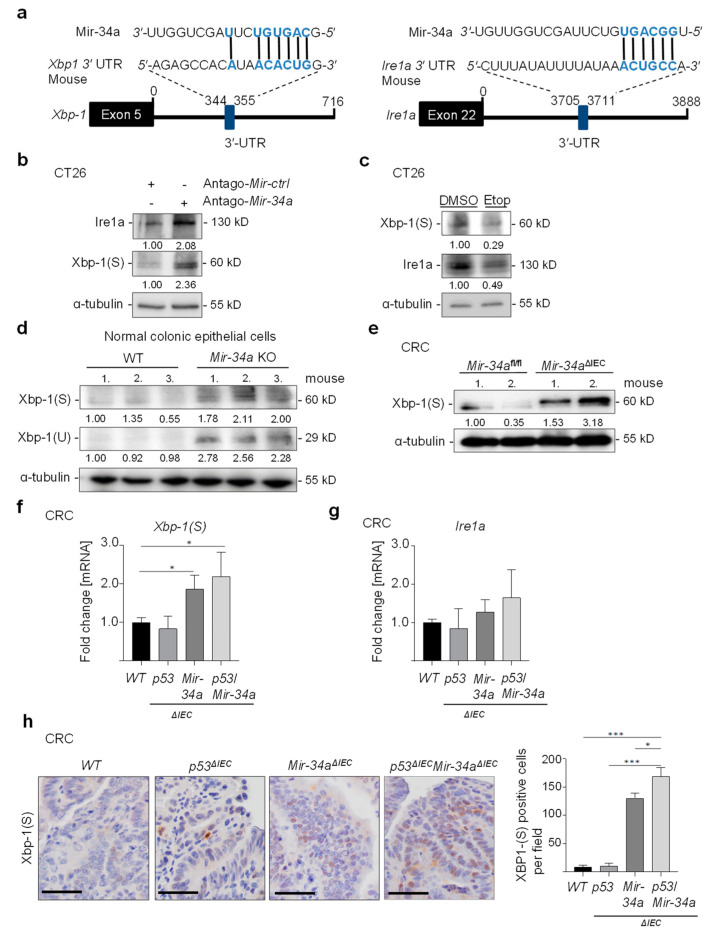
Xbp-1(S)/Ire1a expression in *Mir-34*a-deficient mice. (**a**) Schematic representation of the murine *Xbp-1* and *Ire1a* 3′-UTRs indicating the miR-34a seed matching sequences (SMS). The black vertical bars indicate possible base pairing. (**b**) Western blot analysis of Xbp-1(S) and Ire1a in CT26 cells transfected with antago-Mir-34a or antago-miR negative control oligonucleotides for 48 h. (**c**) Western blot analysis of Xbp-1(S) and Ire1a in CT26 cells after addition of etoposide (20 µM) for 48 h. (**d**) Western blot analysis of Xbp-1(S) and Xbp-1(U) in lysates prepared from colon epithelial cells isolated from WT and *Mir-34a* KO. (**e**) Western blot analysis of XBP-1(S) in lysates prepared from tumors of AOM/DSS-treated *Mir-34a^fl/fl^* and *Mir-34a*^ΔIEC^ mice. (**f**,**g**) qPCR analysis of *Xbp-1(S)* and *Ire1a* in tumor tissues of *Mir-34a*^ΔIEC^/*p53*^ΔIEC^, *Mir-34a*^ΔIEC^, *p53*^ΔIEC^ and WT mice. (**h**) Immunohistochemistry (IHC) detection of Xbp-1(S) in tumor tissues of *Mir-34a^ΔIEC^*/*p53^ΔIEC^, Mir-34a^ΔIEC^*, *p53^ΔIEC^* and WT mice. Quantification of Xbp-1(S) positive cells in CRCs was determined in ≥5 tumors per mouse (n = 2 mice for each genotype). In (**f**–**h**) mean values ± SD (n = 3) are provided. (***) *p* < 0.001, (*) *p* < 0.05.

Inspection of the murine *Xbp-1* and *Ire1a* 3′-UTRs using the TargetSCAN algorithm revealed the presence of conserved Mir-34a seed-matching sequences (SMS) in these mRNAs (Figure 4a). Transfection with Mir-34a-specific antago-miRs, Xbp-1(S), and Ire1a protein levels increased in the CT26 murine cell line (Figure 4b). In addition, treatment of *p53*-proficient CT26 cells with Etoposide resulted in the repression of Xbp-1(S) and Ire1a (Figure 4c). Therefore, the negative regulation of Xbp-1 and Ire1a by p53 and Mir-34a is conserved between humans and mice.

Next, we analyzed Xbp-1 expression in normal colonic epithelium and CRCs of *Mir-34a*-deficient mice that were previously established in our laboratory. The expression of spliced Xbp-1(S) and un-spliced Xbp-1(U) was increased in colonic epithelial cells isolated from *Mir-34a* knockout mice when compared with wild-type mice (Figure 4d). Moreover, we determined the expression of Xbp-1(S) in CRCs derived from mice that were treated six times with AOM (6XAOM) which represents an established mouse model for CRC [27]. *Mir-34a*-deficient CRCs from *Mir-34a*^ΔIEC^ mice displayed robust expression of Xbp-1(S) whereas *Mir-34a*-proficient CRCs only showed marginal expression of Xbp-1(S) (Figure 4e). In this model, expression of *Xbp-1(S)* was significantly increased in CRCs from *Mir-34a^ΔIEC^*/*p53^ΔIEC^* and *Mir-34a^ΔIEC^* mice when compared to CRCs from WT or *p53^ΔIEC^* mice (Figure 4f). mRNA levels of *IRE1A* were also increased in CRCs from *Mir-34a^ΔIEC^* and *p53^ΔIEC^/Mir34a^ΔIEC^* mice (Figure 4g). Moreover, the number of XBP-1(S)-positive cells was increased in CRCs of *Mir-34a^ΔIEC^* and *p53^ΔIEC^/Mir-34a^ΔIEC^* mice when compared to WT and *p53^ΔIEC^* mice (Figure 4h). Taken together, these results suggest that the negative regulation of Xbp-1 and Ire1a by miR-34a is conserved in mice and in vivo.

### 3.5. MiR-34a and IRE1A/XBP-1S Form a Double-Negative Feedback

**Figure 5:**
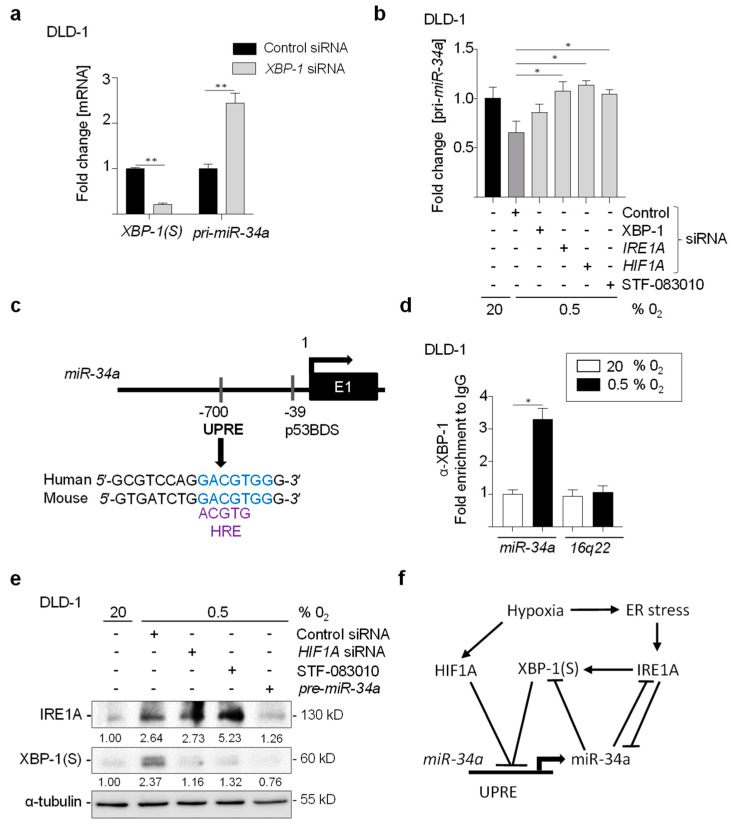
MiR-34a and XBP-1IRE1A/HIF1A form a double-negative feedback. (**a**) qPCR analysis in DLD-1 cells transfected with *XBP-1* or control siRNAs for 24 h, then cultured at 20% O_2_ or 0.5% O_2_ for 72 h. (**b**) qPCR analysis of *pri-miR-34a* in DLD-1 cells transfected with *XBP-1, IRE1A,* or control siRNAs, or treated with STF-083010, for 24 h then cultured at 20% or 0.5% O_2_ for 72 h. (**c**) Map of the human miR-34a genomic region indicating conserved XBP-1(S) binding sites. (**d**) Chromatin immunoprecipitation analysis of XBP-1(S) occupancy at human miR-34a and 16q22 locus served as negative control. (**e**) Western Blot analysis in DLD-1 cells transfected with *HIF1A* or *pre-miR-34a* or control siRNAs, or treated with STF-083010 for 24 h, then cultured at 20% or 0.5% O_2_ for 72 h. (**f**) Model of the coherent feed-forward regulations of and downstream effects. In panel (**a**,**b**,**d**), mean values ± SD (n = 3) are provided. (**) *p* < 0.01, (*) *p* < 0.05.

We observed an increase in *pri-miR-34a* expression after the inactivation of *XBP-1* by siRNAs (Figure 5a). Moreover, down-regulation of XBP-1, *IRE1A,* or *HIF1A* by siRNAs or by inhibition of IRE1A activity by STF-083010 treatment prevented the repression of *pri-miR-34a* by hypoxia in DLD-1 (Figure 5b). Here, we identified a conserved XBP-1 (S) binding site within the sequence upstream of the *miR-34a* transcriptional start site, indicated as UPRE (UPR element) (Figure 5c), which overlaps with the HIF1A-binding site in the *miR-34a* promoter that we had previously characterized [10]. Thus, we hypothesized that XBP-1(S) may directly repress *miR-34a*. Indeed, XBP-1(S) occupancy at the UPRE was detected by chromatin-immunoprecipitation (ChIP) in hypoxic DLD-1 cells (Figure 5d). Moreover, the down-regulation of *HIF1A* by siRNAs did not affect the expression of IRE1A under hypoxia demonstrating that the induction of IRE1A by hypoxia is HIF1A-independent (Figure 5e). Therefore, activation of IRE1A by hypoxia may lead to enhanced expression of XBP-1(S) and repression of *miR-34a* via multiple pathways as depicted in the model shown in (Figure 5f).

### 3.6. Roles of XBP-1(S) and IRE1A in Hypoxia-Induced EMT and Invasion

**Figure 6:**
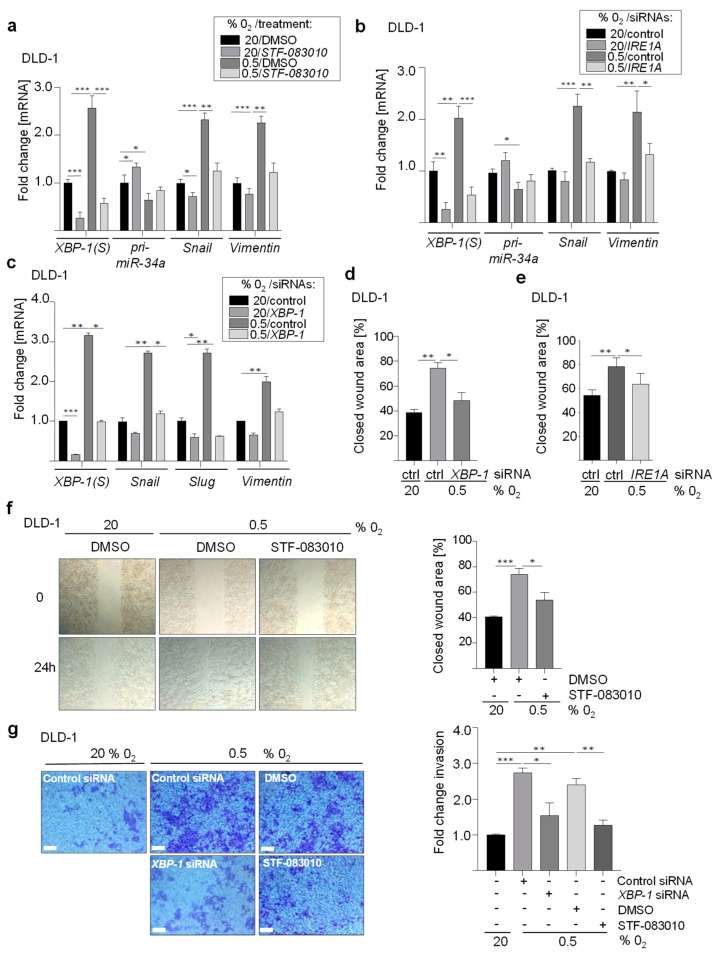
XBP-1(S) and IRE1A are required for hypoxia-induced EMT and invasion. (**a**) qPCR analysis in DLD-1 cells treated with DMSO or STF-083010 (60 µM) for 24 h, then cultured at 20% O_2_ or 0.5% O_2_ for 72 h. (**b**) qPCR analysis in DLD-1 cells transfected with *IRE1A* or control siRNAs and then cultured at 20% O_2_ or 0.5% O_2_ for 72 h. (**c**) qPCR analysis in DLD-1 cells transfected with *XBP-1* or control siRNAs and then cultured at 20% O_2_ or 0.5% O_2_ for 72 h. (**d**) Densitometric representation of the wound-healing assay performed in DLD-1 cells transfected with *XBP-1* or control siRNAs and then cultured at 20% O_2_ or 0.5% O_2_. The normalized wound area was calculated using Image J software. (**e**) Densitometric representation of the wound-healing assay performed in DLD-1 cells transfected with *IRE1A* or control siRNAs and then cultured at 20% O_2_ or 0.5% O_2_. The normalized wound area was calculated using Image J software. (**f**) Densitometric representation and phase-contrast images of the wound-healing assay of DLD-1 treated with DMSO or STF-083010 (60 µM) and cultured at 20% O_2_ or 0.5% O_2_ for 72 h. The normalized wound area was calculated using Image J software. (**g**) Relative invasion in DLD-1 cells transfected with XBP-1 or control siRNAs, or treated with DMSO or STF-083010 (60 µM) and cultured at 20% O_2_ or 0.5% O_2_ for 72 h. In panel (**a**–**g**) mean values ± SD (n = 3) are provided. (***) *p* < 0.001, (**) *p* < 0.01 (*), *p* < 0.05.

Next, we determined whether the miR-34a targets identified here mediate EMT and downstream processes, such as migration and invasion, after exposure to hypoxia. Interestingly, treatment of DLD-1 cells with STF-083010, a specific inhibitor of IRE1A and therefore *XBP-1* mRNA splicing, prevented the induction of the EMT-markers *Snail* and *Vimentin* by hypoxia in DLD-1 cells (Figure 6a).

In addition, down-regulation of *IRE1A* or *XBP-1* by treatment with a single or a pool of siRNAs prevented the induction of *Snail*, *Slug*, *Zeb1,* and *Vimentin* in DLD-1 cells under hypoxia (Figure 6b,c and Figure S2a,b). Moreover, treatment with a pool of siRNAs against *XBP-1* prevented the loss of the epithelial marker *E-cadherin/CDH1* from the outer membrane in DLD-1 cells by hypoxia (Figure S1c,d). In addition, down-regulation of *XBP-1* or *IRE1A* by treatment with a single or a pool of siRNAs also decreased cellular migration, as determined in a scratch-assay (Figure 6d,e and Figure S1e,f). Inhibition of IRE1A by STF-083010 treatment in DLD-1 cells also decreased wound closure (Figure 6f). Moreover, the down-regulation of IRE1A by STF-083010 treatment or *XBP-1* by siRNAs significantly reduced invasion under hypoxia (Figure 6g). Therefore, the activation of IRE1A and XBP-1(S) is necessary for the induction of EMT, migration, and invasion by hypoxia in CRC cells.

### 3.7. IRE1A/XBP-1S Activation Is Necessary for Hypoxia-Induced Metastasis

**Figure 7:**
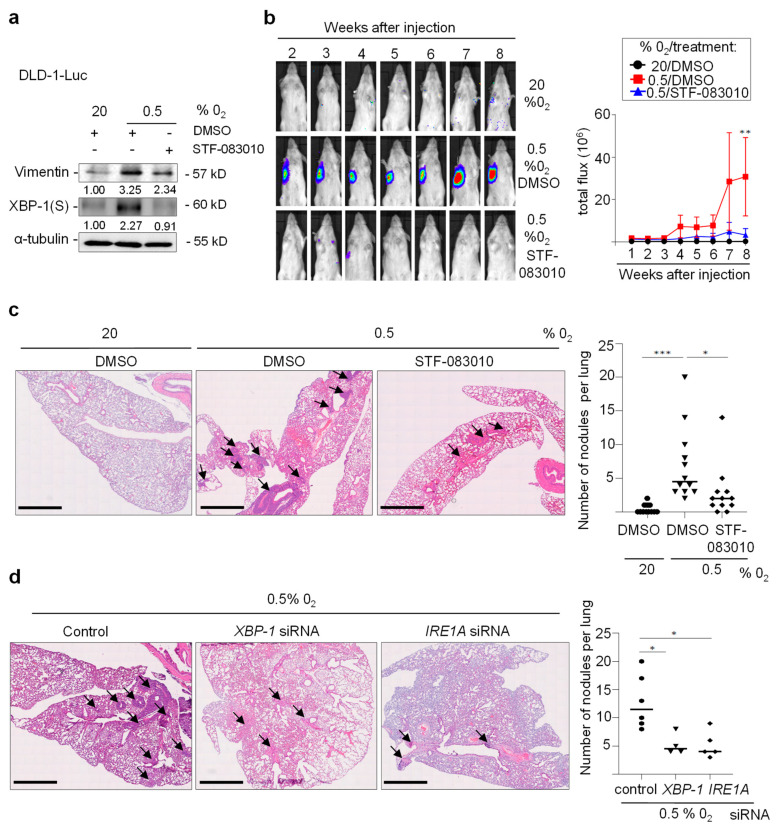
Inhibition of XBP-1(S)/IRE1A decreases lung-metastases formation. (**a**) Western Blot analysis in DLD-1-luc cells treated with STF-083010 for 24 h, then cultured at 20% or 0.5% O_2_ for 30 h. (**b**–**c**) DLD-1-luc cells treated with DMSO or STF-083010 (60 µM) and cultured at 0.5% O_2_ for 30 h, subsequently injected into the tail veins of immune-compromised NOD/SCID mice (n = 12), and followed by noninvasive bioluminescence imaging for 8 weeks. (**b**) Representative examples of bioluminescence imaging and quantification of noninvasive imaging at the indicated time points. Bioluminescence signals are presented as “total flux.” (**c**) Representative images of lungs, arrows indicate metastatic nodules (left panel) and quantification of metastatic tumor nodules in the lung per mouse 8 weeks after tail-vein injection (right panel). (**d**) DLD-1 cells transfected with *XBP-1*, *IRE1A,* or control siRNAs for 24 h, then cultured at 0.5% O_2_ for 30 h, subsequently injected into the tail veins of immune-compromised NOD/SCID mice (n = 6), and followed by noninvasive bioluminescence imaging for 8 weeks. Representative images of lungs, arrows indicate metastatic nodules (left panel); and quantification of metastatic tumor nodules in the lung per mouse 8 weeks after tail-vein injection (right panel). In (**b**–**d**) mean values ± SD (n = 3) are provided. (***) *p* < 0.001, (**) *p* < 0.01, (*) *p* < 0.05. arrows indicate metastatic nodules.

We had previously demonstrated that pretreatment of DLD-1 CRC cells with 0.5% O_2_ for 48 h and their subsequent injection into tail veins of NOD/SCID mice resulted in the formation of lung metastases, whereas untreated DLD-1 cells do not form lung metastases [10]. Furthermore, ectopic miR-34a expression prevents lung metastasis formation [28]. Therefore, we determined whether inhibition of the IRE1A/XPB1 pathway is sufficient to prevent hypoxia-induced metastasis. Pre-treatment of DLD-1 cells stably expressing *luciferase* with STF-083010 significantly reduced the expression of the mesenchymal marker Vimentin and blocked XBP1(S) expression under hypoxia (Figure 7a). Importantly, pretreatment of these cells with STF-083010 efficiently inhibited hypoxia-induced lung metastases formation after i.v. injection into NOD/SCID mice (Figure 7b,c). Moreover, siRNA-mediated silencing of *XBP-1* or *IRE1A* prior to exposure of DLD-1 cells to hypoxia significantly reduced the number of metastatic tumor nodules that these cells formed after injection into NOD/SCID mice (Figure 7d). Taken together, these results show that activation of the IRE1A/XBP-1(S) is required for the hypoxia-induced formation of metastases by CRC cells.

### 3.8. XBP-1(S) Mediates Hypoxia-Induced Chemo-Resistance and Autophagy

**Figure 8:**
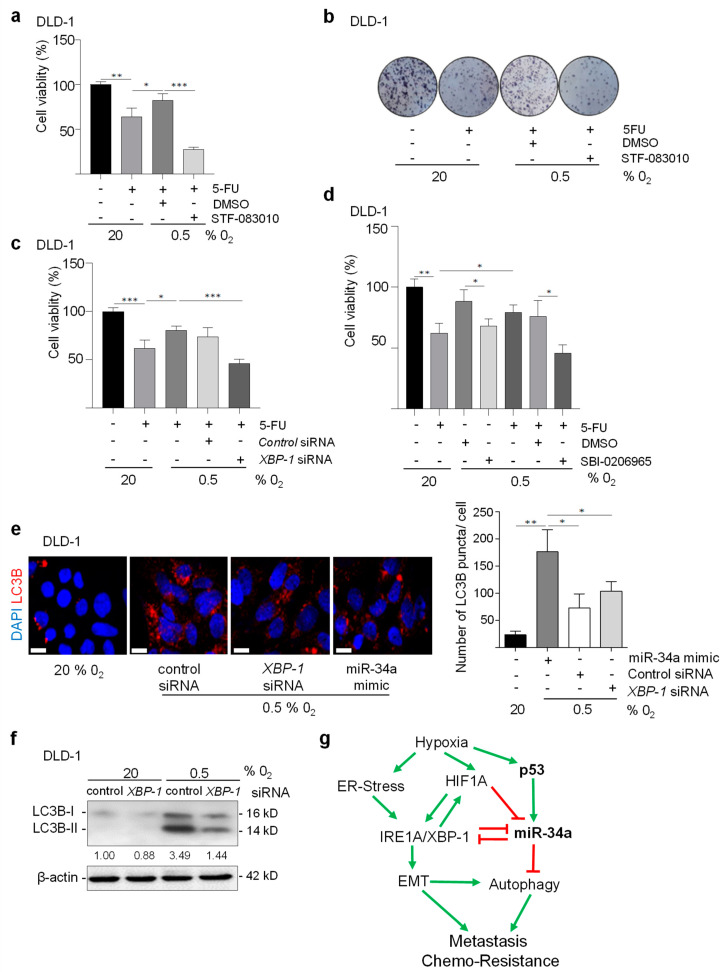
Induction of XBP-1(S) is required for hypoxia-induced autophagy and chemo-resistance. (**a**) MTT assay of DLD-1 cells treated with DMSO or STF-083010 for 24 h at 20.0% O_2_, then exposed to 0.5% O_2_ for 48 h, and subsequently treated with or without 5-FU for 72 h. (**b**) Representative examples of crystal violet staining of DLD-1 cells treated with DMSO or STF-083010 for 24 h at 20.0% O_2_, then exposed to 0.5% O_2_ for 48 h, and subsequently treated with or without 5-FU for 72 h. (**c**) MTT assay of DLD-1 cells transfected with *XBP-1* or control siRNAs for 24 h at 20.0% O_2_, then exposed to 0.5% O_2_ for 48 h, and subsequently treated with or without 5-FU for 72 h. (**d**) MTT assay of DLD-1 cells treated with DMSO or SBI-0206965 (10 µM) for 24 h hours at 20.0% O_2_, then exposed to 0.5% O_2_ for 48 h, and subsequently treated with or without 5-FU for 72 h. (**e**) Indirect immunofluorescence detection of LC3B in DLD-1 cells transfected with *XBP-1*, control siRNAs or *pre-miR-34a* for 24 h then exposed to 0.5% O_2_ for 48 h. (**f**) Western blot analysis of LC3B in DLD-1 cells transfected with XBP-1 or control siRNAs exposed to 20% or 0.5% O_2_ for 48 h. (**g**) The HIF1A/IRS1A/XBP1/miR-34a regulatory circuit: Schematic model summarizing the findings of this study. Green arrows represent stimulatory and red lines inhibitory effects. Loss or mutation of p53/miR-34a in CRCs may enhance the indicated, pro-tumorigenic processes. In (**a**,**c**–**e**) mean values ± SD (n = 3) are provided. (***) *p* < 0.001, (**) *p* < 0.01, (*) *p* < 0.05.

We have previously demonstrated that hypoxia mediates chemo-resistance towards 5-FU by suppressing *miR-34a* expression [10]. Importantly, p53 and miR-34a determine the response of tumor cells to 5-FU treatment under hypoxia [10]. Notably, *p53*-deficient cells were more resistant to 5-FU under hypoxia. Therefore, we determined whether the miR-34a targets identified here modulate the cellular response of DLD-1 cells to 5-FU under hypoxia. DLD-1 cells express mutant p53 [29] and should therefore not induce miR-34a expression after hypoxia. Interestingly, *XBP-1* inhibition by siRNAs or treatment with STF-083010 significantly reduced the viability of DLD-1 cells treated with 5-FU (Figure 8a–c and Figure S2a). Therefore, the up-regulation of XBP-1 and IRE1A is required for hypoxia-mediated resistance towards 5-FU.

It has been shown previously, that hypoxia-induced autophagy contributes to chemo-resistance [30,31,32]. Consistently, the resistance of DLD-1 cells to 5-FU observed at 0.5% O_2_ was reduced by treatment with SBI-0206965, a highly selective autophagy kinase ULK1 inhibitor or with chloroquine, which inhibits autophagy (Figure 8d and Figure S2b). Since XBP-1 has been linked to the induction of autophagy [33], we asked whether XBP-1 mediates the induction of autophagy by hypoxia in CRC cells. Indeed, the knockdown of XBP-1 in DLD-1 cells significantly reduced the hypoxia-induced accumulation of LC3B-II (Figure 8e) and the number of cells positive for LC3B puncta, which indicates the formation of autophagosomes in DLD-1 cells under hypoxic conditions (Figure 8f). Notably, miR-34a is a known inhibitor of autophagy as it targets multiple factors implicated in autophagy [34,35]. Indeed, treatment of DLD-1 cells with *pre-miR-34a* oligonucleotides significantly reduced the number of LC3B puncta-positive cells under hypoxic conditions (Figure 8e). Moreover, ectopic expression of *pri-miR-34a* in SW480 cells resulted in the reduction of LC3B puncta-positive cells and decreased LC3B-II accumulation (Figure S2c,d). Furthermore, treatment with Mir-34a-specific antagomiRs induced the transition of LC3B-I to LC3B-II in murine CT26 cells (Figure S2e). Taken together, our findings show that during the response to hypoxia, the induction of XBP-1(S) is required for autophagy-induced chemo-resistance. In addition, repression of *miR-34a* by HIF1A and/or XBP-1 is presumably also important for autophagy in this context, as miR-34a would otherwise inhibit autophagy by targeting multiple components of the autophagic process.

## 4. Discussion

The interplay between HIF-1A and p53 may serve as a critical determinant of cancer invasion and metastasis under hypoxic conditions, and a potential determinant of therapeutic outcomes for colorectal cancer patients [8]. At present, the interactions between p53 and HIF-1A are not completely understood. The scenario is made more complex by the substantial number of target genes of both factors that represent components of numerous signaling pathways and biochemical processes, which control tumor cell survival. Besides the p53-mediated regulation of genes encoding proteins, p53-induced microRNAs have emerged as important effectors of p53 functions. Importantly, miR-34a represents the miRNA, which is induced by p53 most profoundly in all tested cell types, implying outstanding importance of miR-34a among all p53-induced miRNAs [36]. We have previously shown that *PPP1R11/INH3* is subject to a feed-forward regulation by HIF1A and miR-34a, which mediates its induction under hypoxic conditions. Furthermore, Inh3 expression was required for induction of EMT, invasion, and migration by hypoxia in p53-deficient colorectal cell lines [10]. Here, we characterized novel regulators and effectors of miR-34a. We found that the decision of tumor cells to undergo EMT or MET in response to hypoxia is mediated by a regulatory network involving HIF1A, p53, miR-34a, and its new targets, IRE1A and XBP-1 (see also Figure 8g for a summary model). We found that XBP-1(S)/IRE1A and miR-34a form a double-negative feedback regulatory loop, wherein miR-34a represses XBP-1 and IRE1A under normoxia, in *TP53*-proficient CRC cells. In *TP53-*defective CRC cells HIF-1A, XBP-1(S), and IRE1A may cooperate to repress and degrade miR-34a under hypoxia. The decrease in miR-34a levels may further increase the levels of IRE1A, which activates XBP-1(S). Subsequently, the resulting activation of XBP-1(S) mediates hypoxia-induced EMT, migration, invasion and ultimately contributes to metastasis.

The negative regulation of XBP-1S by the tumor suppressive p53/miR-34a axes is in line with an oncogenic/pro-tumorigenic function of the IRE1A/XBP-1(S) axes. This is also consistent with its documented role in the c-MYC network [37,38]. XBP-1(S) activation and the subsequent increase in protein folding capacities represent a central facilitator of tumor cell growth and tumor expansion/migration. Its negative regulation by the p53/miR-34a axes is therefore presumably central to the tumor suppressor functions of p53 and the miR-34 family. Here, we found that the inactivation of XBP-1(S)/IRE1A prevents the hypoxia-induced formation of lung-metastases in mice. Our findings imply that activation of the IRE1A/XBP1S pathway is necessary for tumor cells to acquire a mesenchymal/invasive phenotype under hypoxia.

Previously, we showed that *miR-34a* is directly repressed by HIF-1A under hypoxic conditions in the *p53*-deficient cells [10]. Here, we further demonstrated that XBP-1(S) enhances the repression of the tumor-suppressive *miR-34a* by HIF-1A. This new mechanism is in accordance with previous works showing that XBP-1(S) plays a key role in cancer cell survival under hypoxia [21,22]. Notably, XBP-1 and HIF-1A co-occupy several well-known HIF-1A targets, and XBP-1 depletion down-regulated HIF-1A targets under hypoxic conditions [21]. In addition, XBP-1 co-localizes with hypoxia markers in tumors and the loss of XBP-1 increases the sensitivity of tumor cells to hypoxia-induced apoptosis and inhibits tumor growth [22], implicating XBP-1(S) as a critical survival factor under hypoxic conditions. Moreover, our results also revealed that the hypoxia-induced XBP-1/IRE1A activation is essential for hypoxia-mediated resistance against 5-FU through the induction of autophagy. Thereby, *p53* and/or *miR-34a* inactivation, which is commonly found in CRC and other tumors, may promote tumor cell survival. Altogether, our results reveal a new p53/miR-34a/XBP-1/IRE1A regulatory circuitry that may play a crucial role during hypoxia-driven tumor cell survival and metastasis. In addition, up-regulation of miR-34a may prevent chemo-resistance by targeting XBP-1/IRE1A. Restauration of miR-34a function by delivery of miR-34a mimics to tumor cells using several different approaches is being clinically evaluated [39]. In the future, miR-34a restoration may be an attractive therapeutic strategy to treat cancer or an alternative approach to overcome chemo-resistance.

## 5. Conclusions

In summary, HIF-1A, p53-induced miR-34a, and the IRE1A-XBP-1(S) pathway form a double-negative feedback regulatory loop under hypoxic conditions, wherein activation of HIF-1A/IRE1A induces XBP-1(S) by repressing *miR-34a* to promote EMT, metastasis, autophagy and chemo-resistance in colorectal cancer cells that have undergone inactivation of p53.

## Data Availability

Data is contained within the article and supplementary material.

## Supplementary Materials

The following supporting information can be downloaded at: https://www.mdpi.com/article/10.3390/cancers15041143/s1, Figure S1. (a) qPCR analysis in DLD-1 cells transfected with XBP-1 or control siRNAs Pool. (b) qPCR analysis in DLD-1 cells transfected with XBP-1 or control siRNAs Pool and then cultured at 20% O_2_ or 0.5% O_2_ for 72 h. (c,d) Indirect immunofluorescence detection and western blot analysis of E-cadherin/CDH1 in DLD-1 cells transfected with XBP-1 or control siRNAs Pool and then cultured at 20% O_2_ or 0.5% O_2_. (e,f) Densitometric representation and of the wound-healing assay done in DLD-1 cells transfected with XBP-1 or control siRNAs Pool and then cultured at 20% O_2_ or 0.5% O_2_. The normalized wound area was calculated using Image J software. In panel (a), (b), and (f) mean values ± SD (n = 3) are provided. (***) *p* < 0.001, (**) *p* < 0.01, (*) *p* < 0.05. Figure S2. (a) MTT assay of DLD-1 cells transfected with XBP-1 or control siRNAs Pool for 24 h at 20.0% O_2_, then exposed to 0.5% O_2_ for 48 h, and subsequently treated with or without 5-FU for 72 h. (b) MTT assay of DLD-1 cells treated with DMSO or chloroquine (20 μM) for 24 h at 20.0% O_2_, then exposed to 0.5% O_2_ for 48 h, and subsequently treated with or without 5-FU for 72 h. (c,d) Indirect immunofluorescence detection and western blot analysis of LC3B in SW480 cells harboring a pRTR/pri-miR-34a vector exposed to Doxycycline (DOX) for 48 h. (e) Western blot analysis of LC3B in CT26 cells transfected with antago-miR-34a or antago-miR control oligonucleotides for 48 h. In panel (a), (b) mean values ± SD (n = 3) are provided. (***) *p* < 0.001, (**) *p* < 0.01, (*) *p* < 0.05. Figure S3. Source data of Western blot analyses. Table S1: Oligonucleotides used for quantitative real-time PCR analyses. Table S2: List of antibodies. Table S3: Oligonucleotides used for qChIP. Table S4: Oligonucleotides used for cloning and mutagenesis of *XBP-1* and *IRE1A* 3’UTRs. Table S5: Vectors used in this study. References [40,41,42] are cited in the supplementary materials.
