# Supplementary material for: miR-34a and IRE1A/XBP-1(S) Form a Double-Negative Feedback Loop to Regulate Hypoxia-Induced EMT, Metastasis, Chemo-Resistance and Autophagy
Source: Cancers (Basel). 2023 Feb 10;15(4):1143. doi: 10.3390/cancers15041143 (PMC9954576; doi:10.3390/cancers15041143)
Supplement: Supplementary file 1 [file cancers-15-01143-s001.zip › cancers-2158458-supplementary.pdf]

Supplementary Information

miR-34a and IRE1A/XBP-1(S) Form a Double-Negative Feedback Loop to Regulate Hypoxia-Induced EMT, Metastasis, Chemo-Resistance and Autophagy

Nassim Bouznad, Matjaz Rokavec, Meryem Gülfem Öner and Heiko Hermeking

Inventory of Supplementary Information

- **Supplemental Figure S1**
- **Supplemental Figure S2**
- **Supplemental Figure S3**: Uncropped Western blot membranes
- **Supplemental Table S1**: Oligonucleotides used for qPCR
- **Supplemental Table S2**: List of antibodies
- **Supplemental Table S3**: Oligonucleotides used for qChIP
- **Supplemental Table S4**: Oligonucleotides used for cloning and mutagenesis of *XBP-1* and *IRE1A* 3'-UTRs
- **Supplemental Table S5**: Vectors used in this study

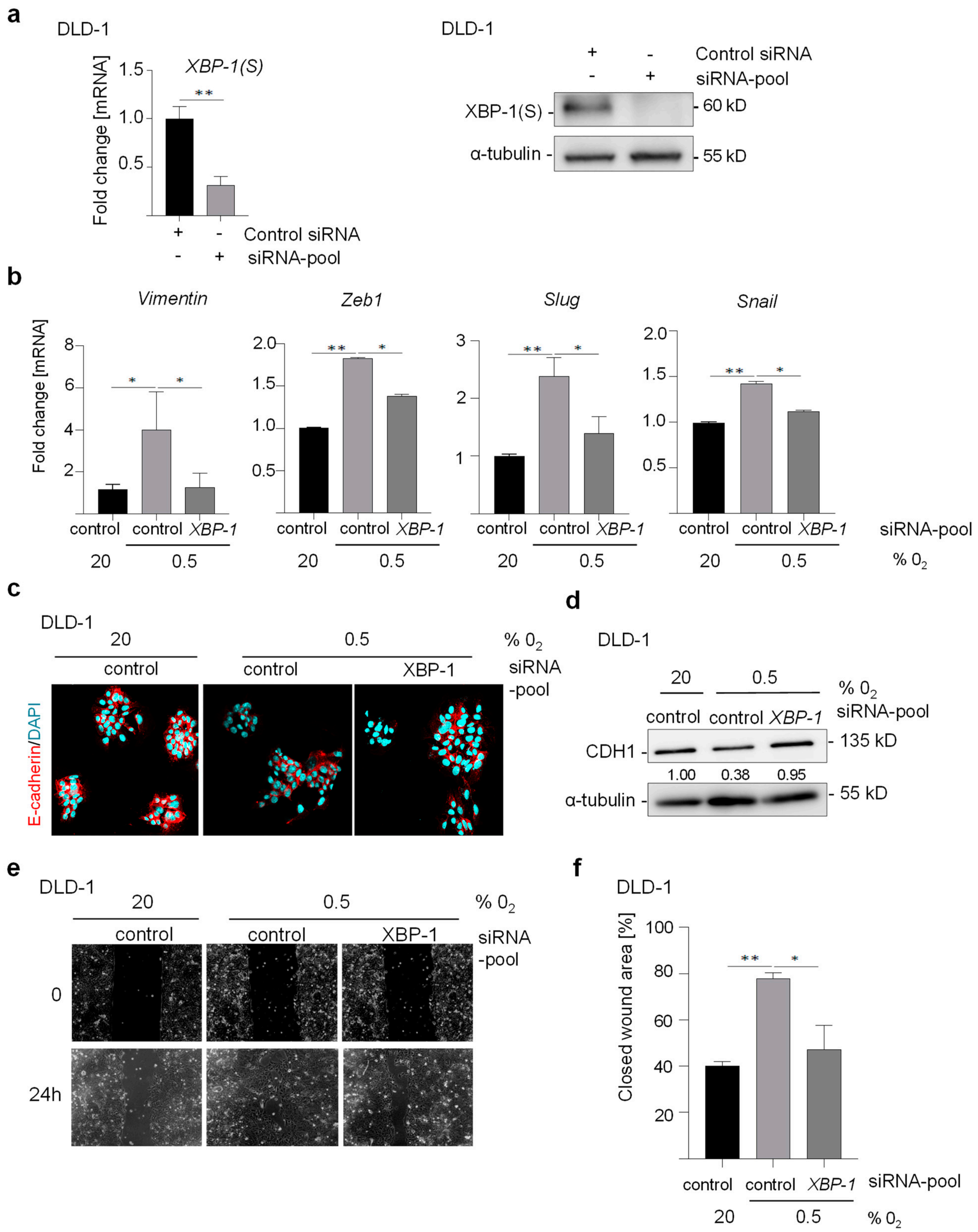

Figure S1. (a) qPCR analysis in DLD-1 cells transfected with *XBP-1* or control siRNAs Pool. **(b)** qPCR analysis in DLD-1 cells transfected with *XBP-1* or control siRNAs Pool and then cultured at 20% O_2 or 0.5% O_2 for 72 hours. **(c-d)** Indirect immunofluorescence detection and western blot analysis of *E-cadherin/*CDH1 in DLD-1 cells transfected with *XBP-1* or control siRNAs Pool and then cultured at 20% O_2 or 0.5% O_2. **(e-f)** Densitometric representation and of the wound-healing assay done in DLD-1 cells transfected with *XBP-1* or control siRNAs Pool and then cultured at 20% O_2 or 0.5% O_2. The normalized wound area was calculated using Image J software. In panel **(a)**, **(b)**, and **(f)** mean values ± SD (n=3) are provided. (***) $p < 0.001$, (**) $p < 0.01$, (*) $p < 0.05$.

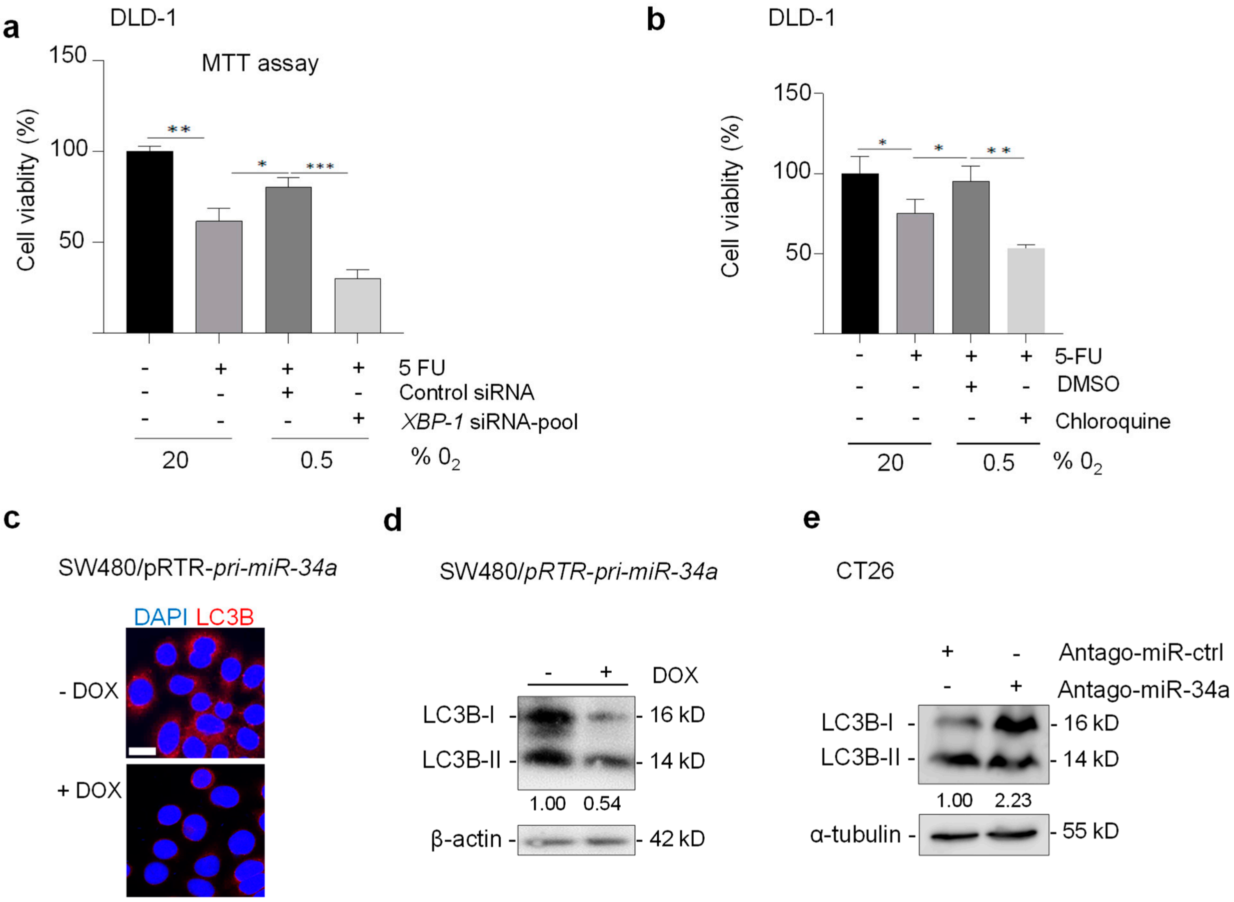

Figure S2. (a) MTT assay of DLD-1 cells transfected with *XBP-1* or control siRNAs Pool for 24hours at 20.0% O_2, then exposed to 0.5% O_2 for 48 hours, and subsequently treated with or without 5-FU for 72 hours. **(b)** MTT assay of DLD-1 cells treated with DMSO or chloroquine (20 μM) for 24h hours at 20.0% O_2, then exposed to 0.5% O_2 for 48 hours, and subsequently treated with or without 5-FU for 72 hours. **(c-d)** Indirect immunofluorescence detection and western blot analysis of LC3B in SW480 cells harboring a pRTR/pri-miR-34a vector exposed to Doxycycline (DOX) for 48h. **(e)** Western blot analysis of LC3B in CT26 cells transfected with antago-miR-34a or antago-miR control oligonucleotides for 48 hours. In panel **(a)**, **(b)** mean values ± SD (n=3) are provided. (***) p< 0.001, (**) p< 0.01, (*) p< 0.05.

Figure S3. Source data of Western blot analyses

Related to Figure 1b

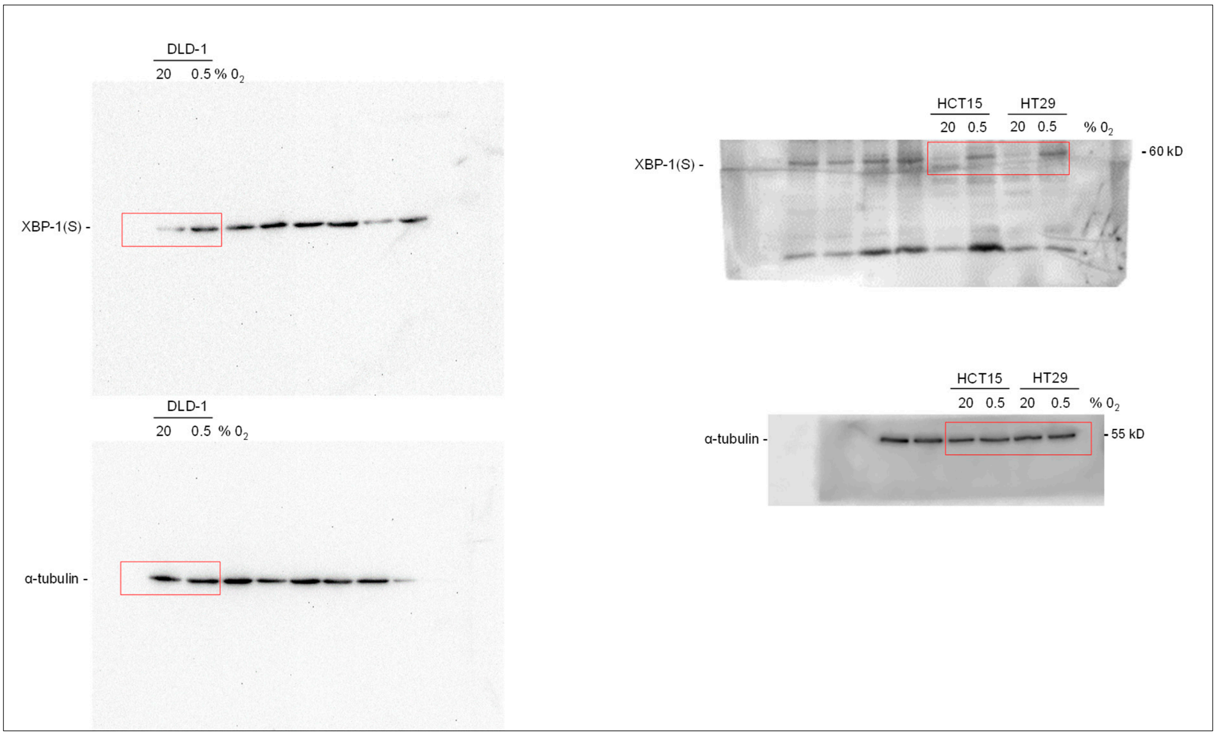

Related to Figure 1f

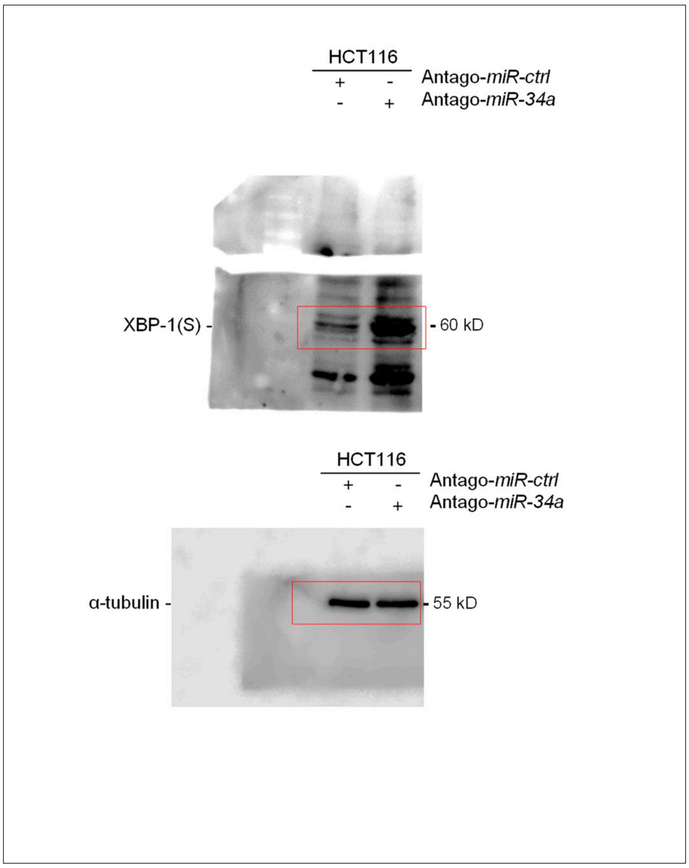

Related to Figure 1h

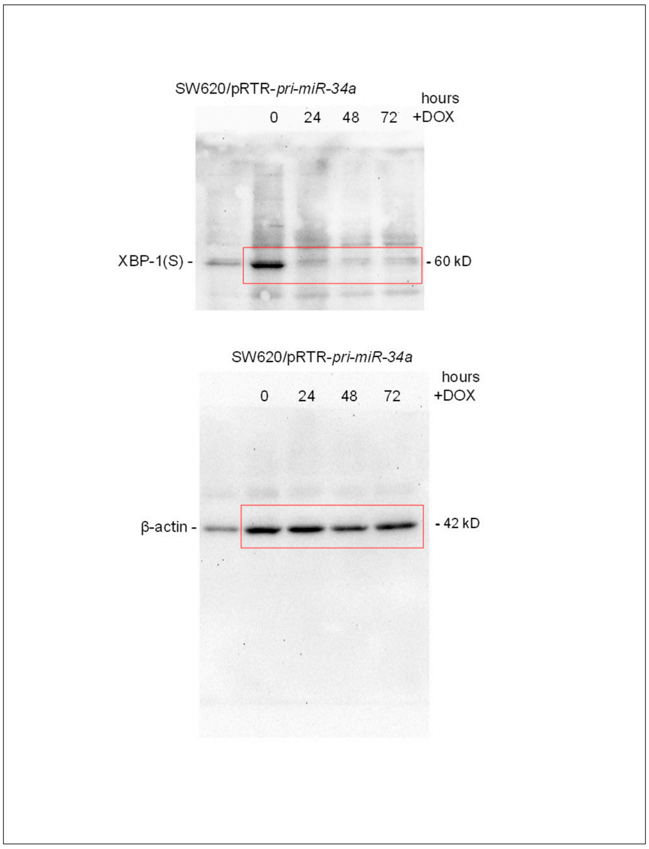

Related to Figure 1i

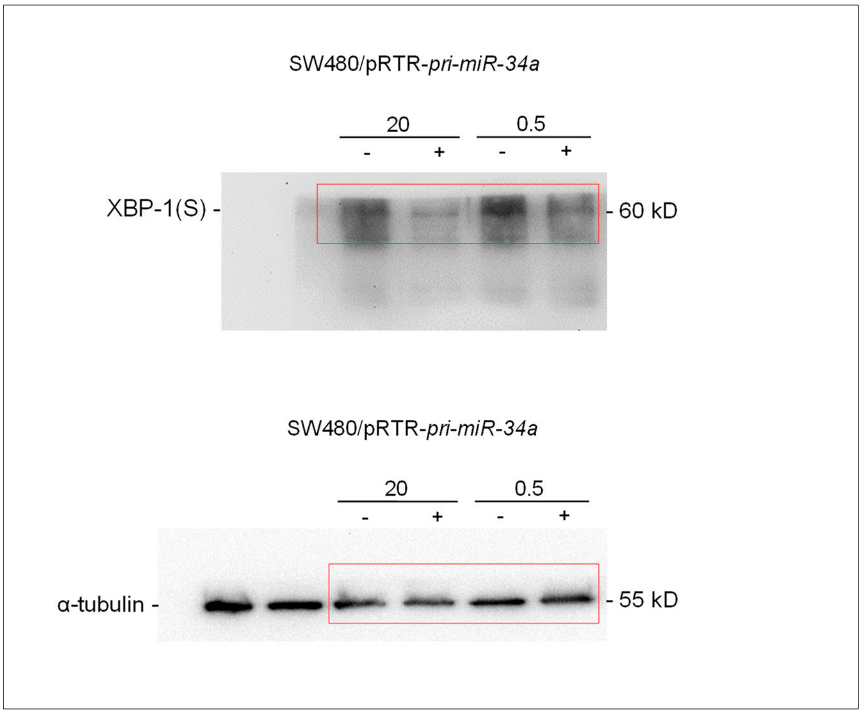

Related to Figure 2a

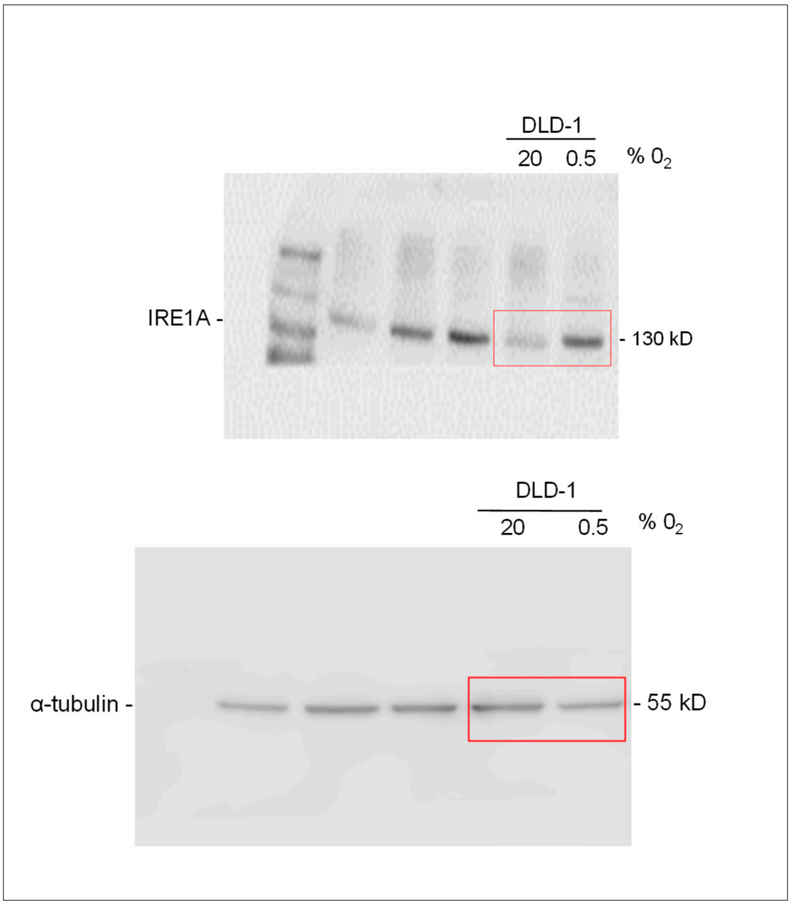

Related to Figure 2c

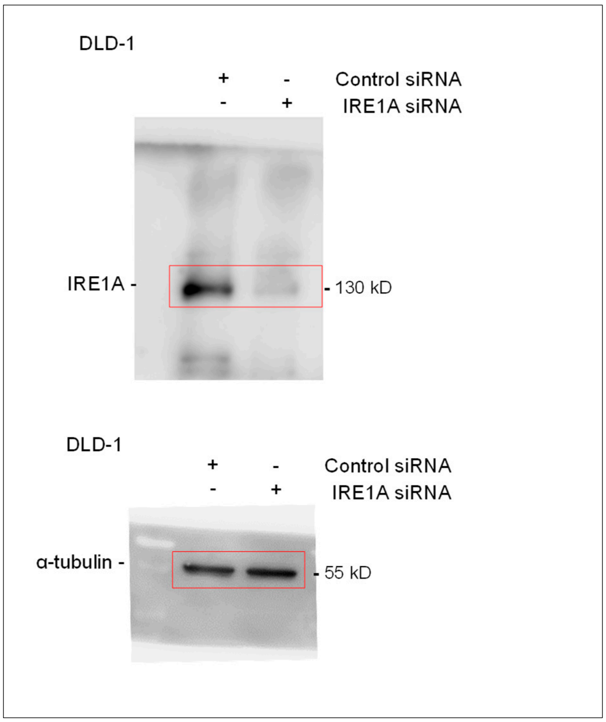

Related to Figure 2d

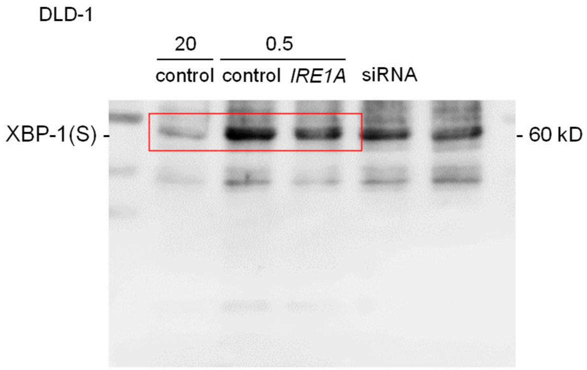

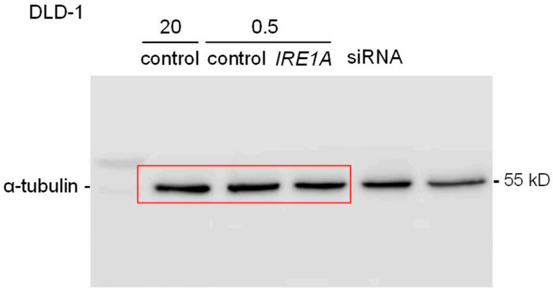

Related to Figure 2e

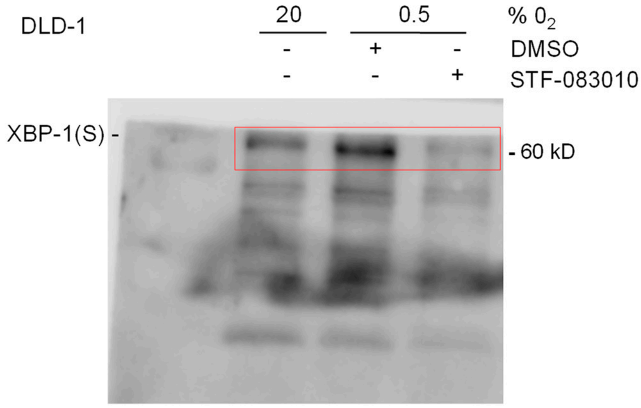

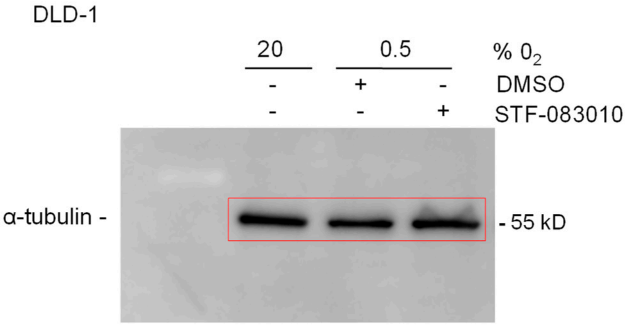

Related to Figure 2i

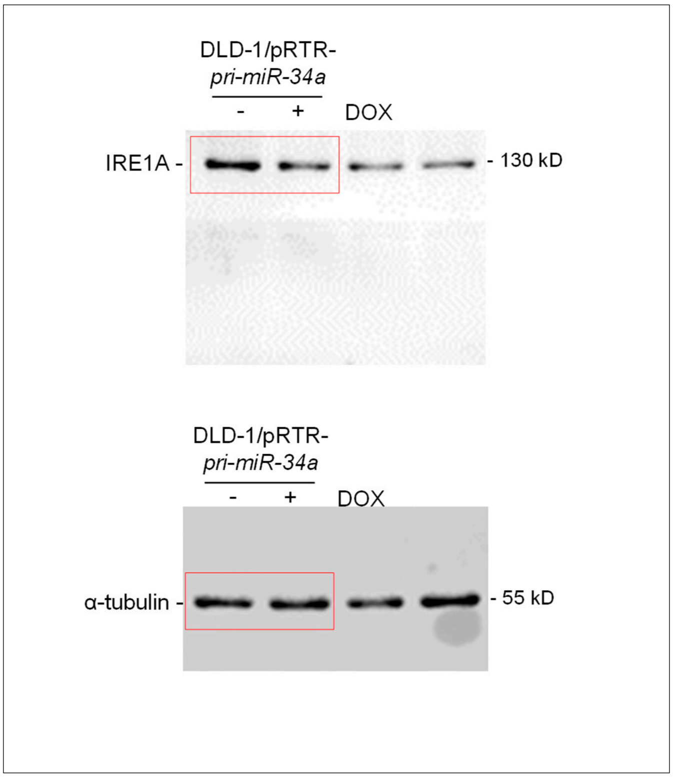

Related to Figure 2j

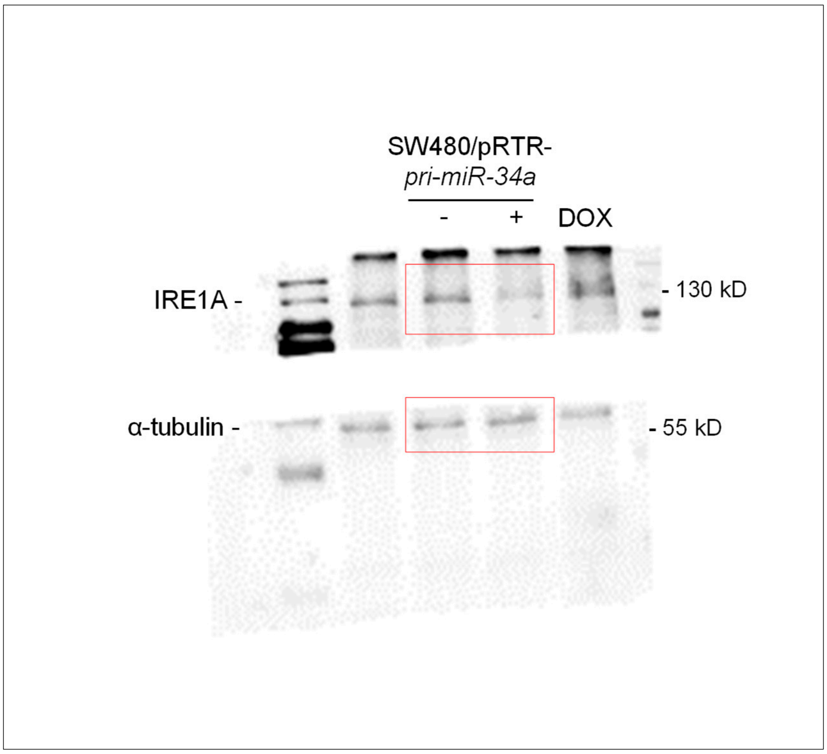

Related to Figure 3a

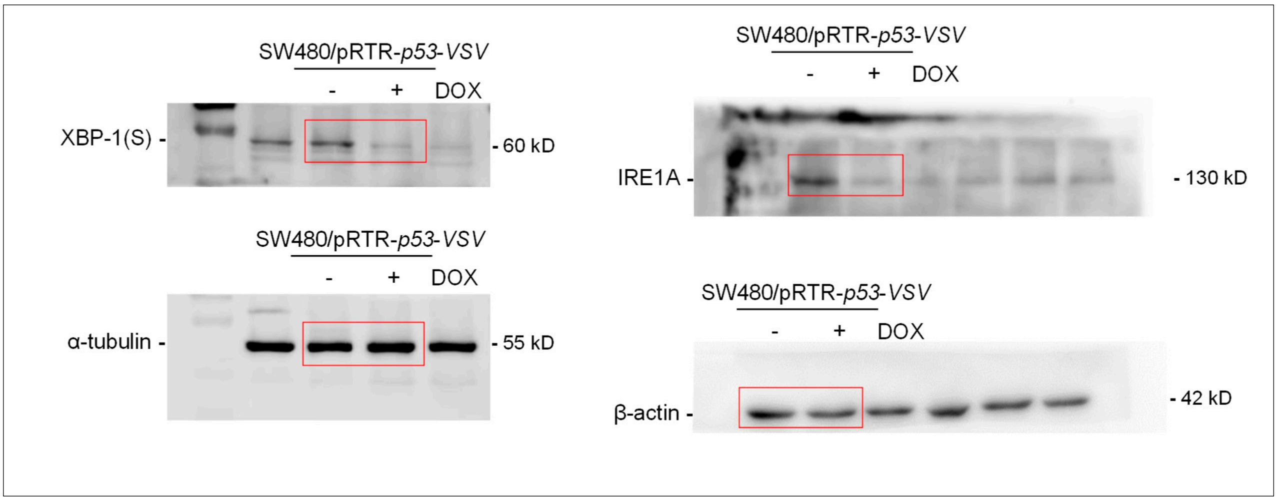

Related to Figure 3c

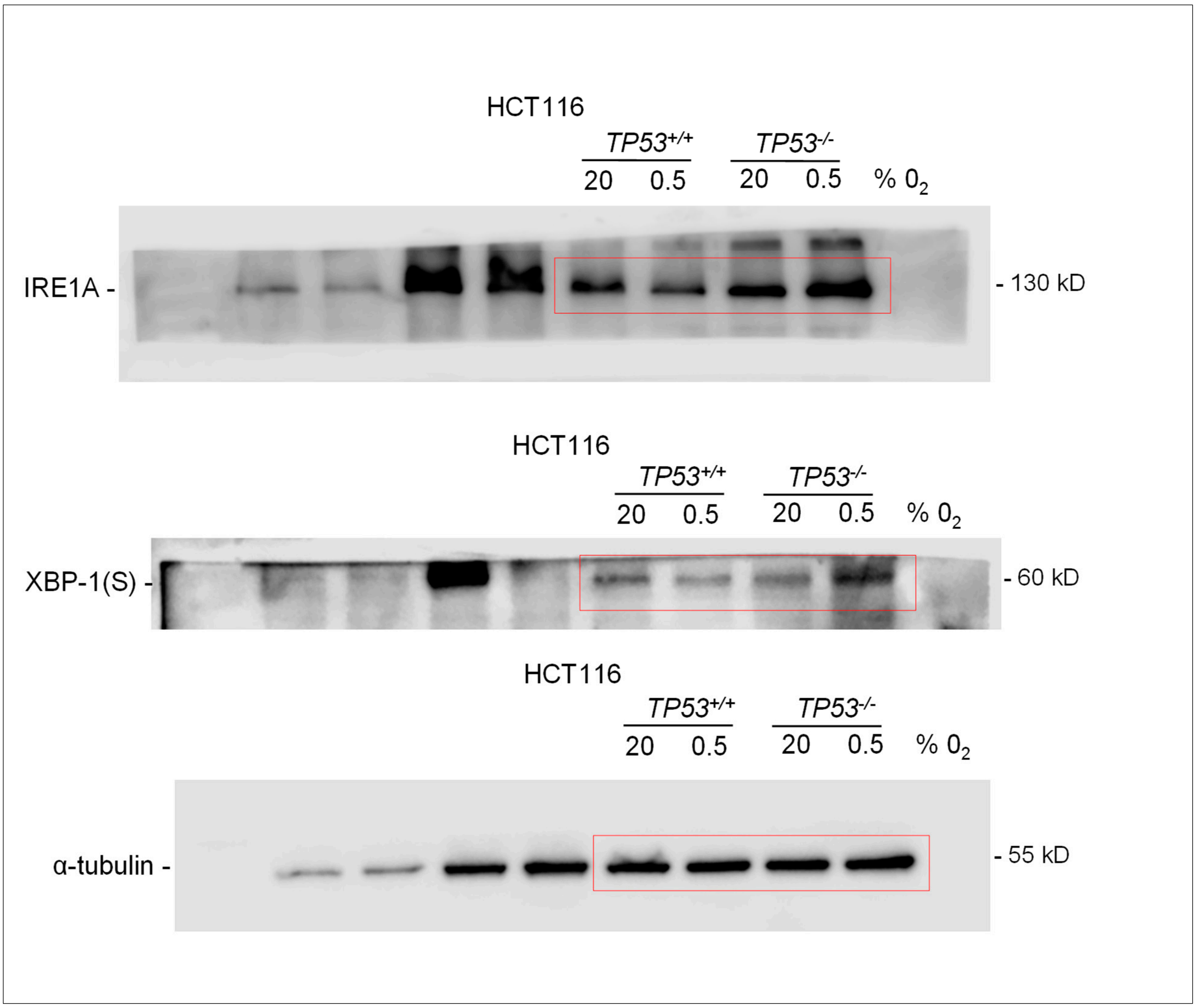

Related to Figure 3e

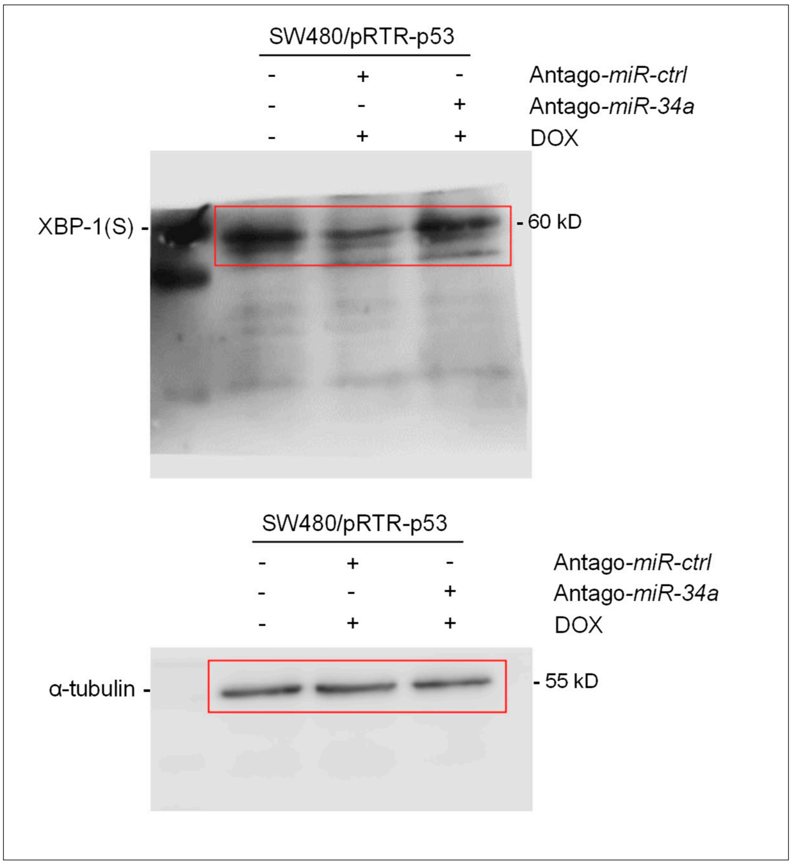

Related to Figure 3f

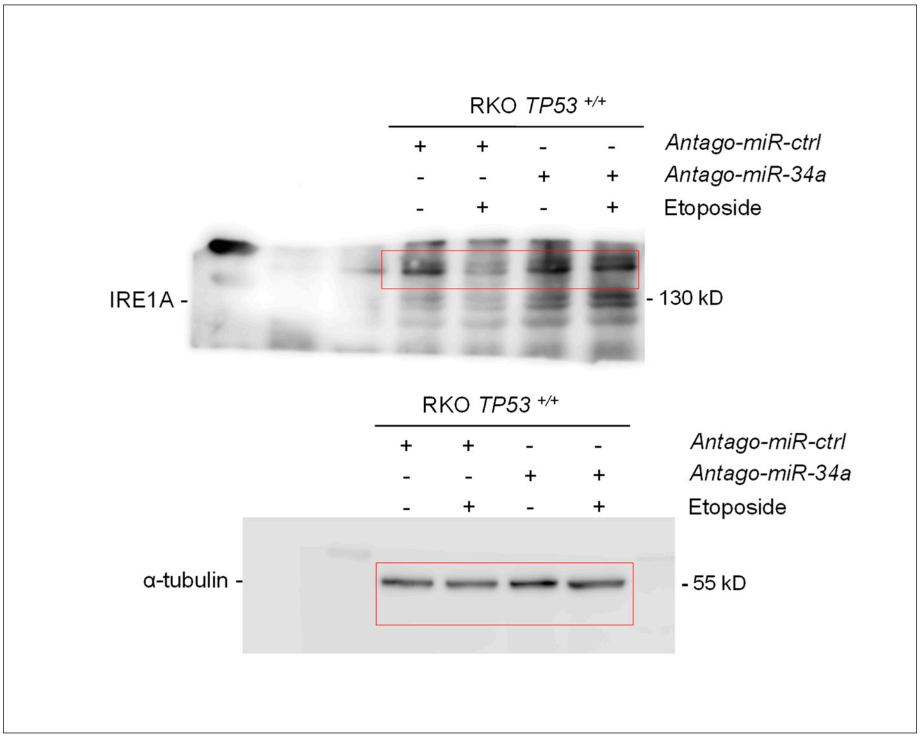

Related to Figure 4b

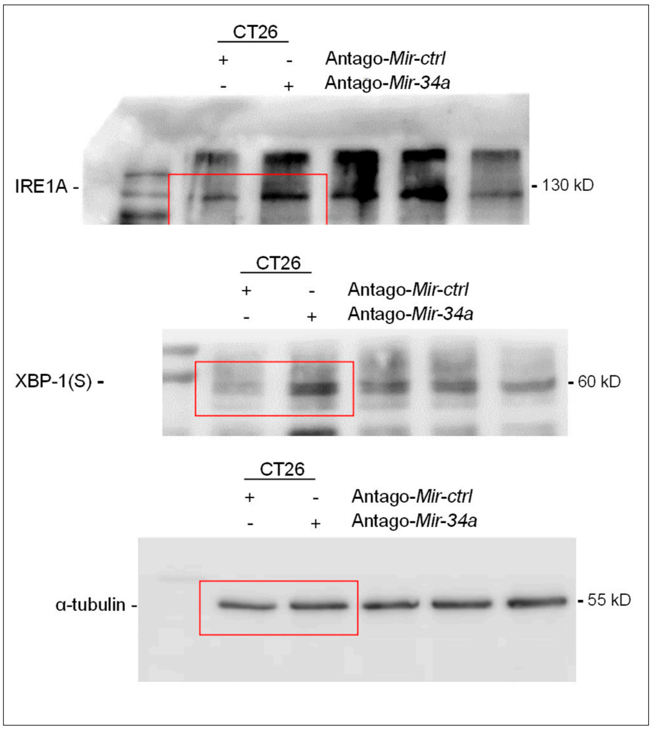

Related to Figure 4c

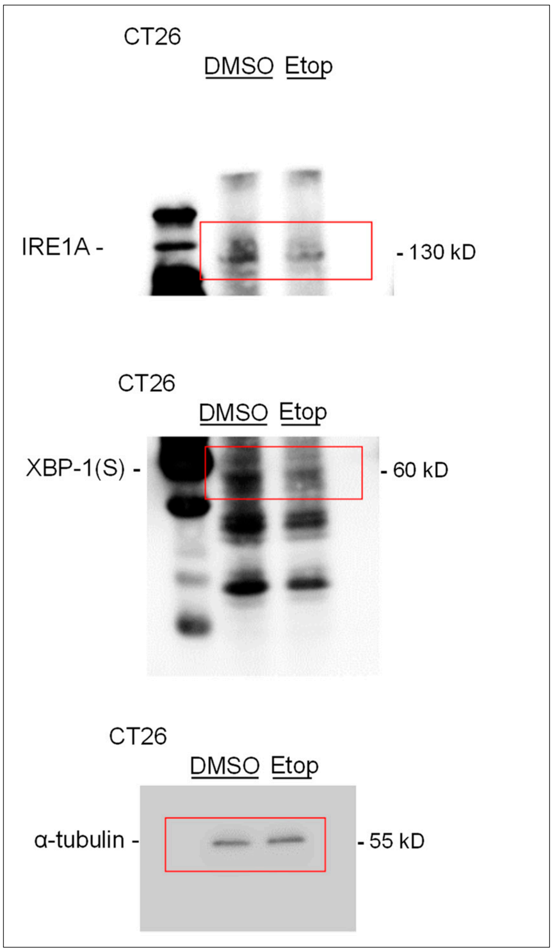

Related to Figure 4d

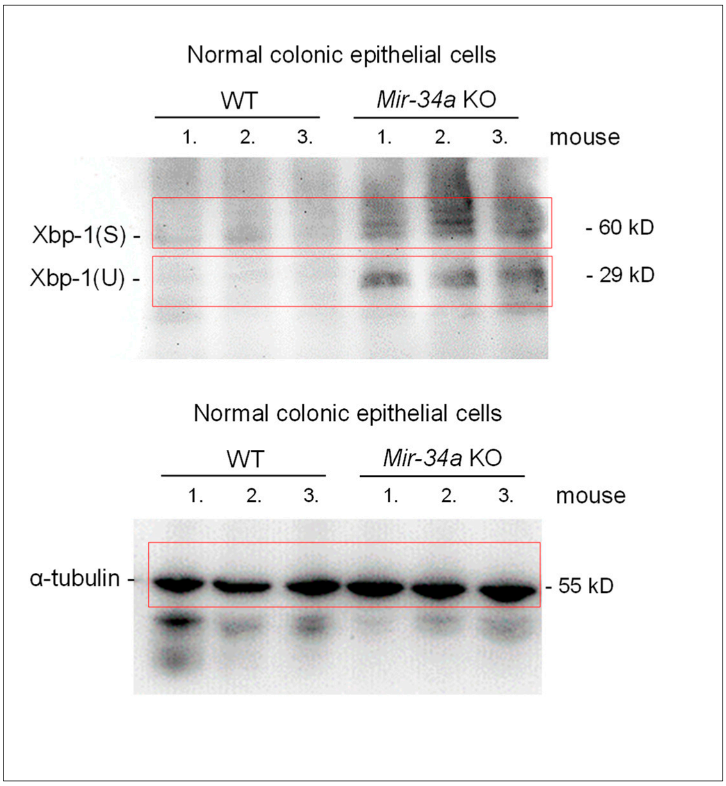

Related to Figure 4e

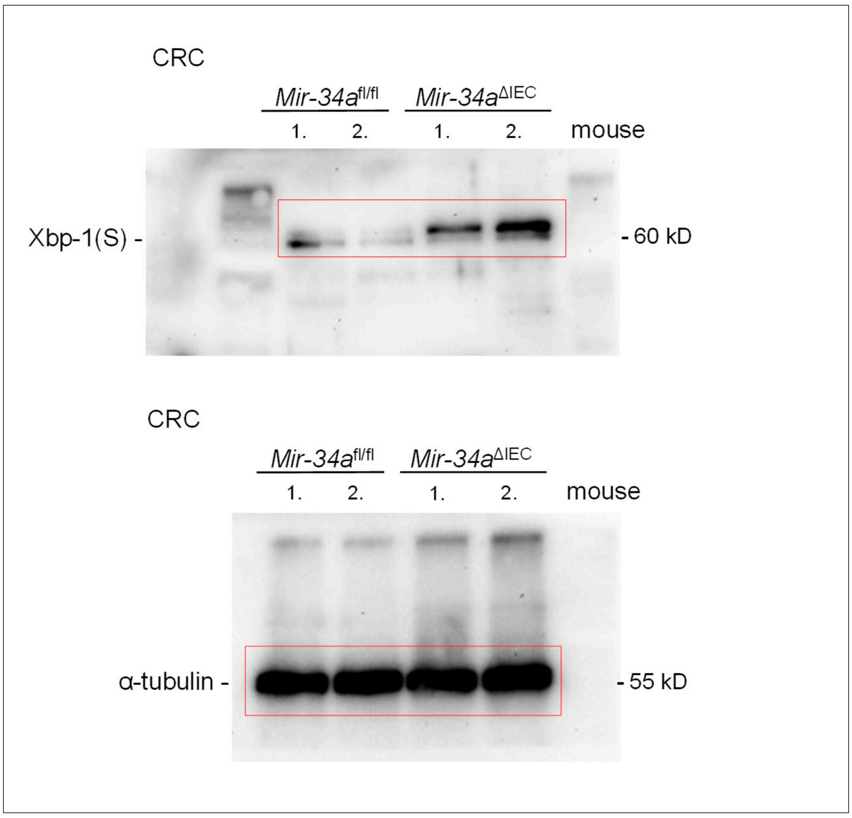

Related to Figure 5e

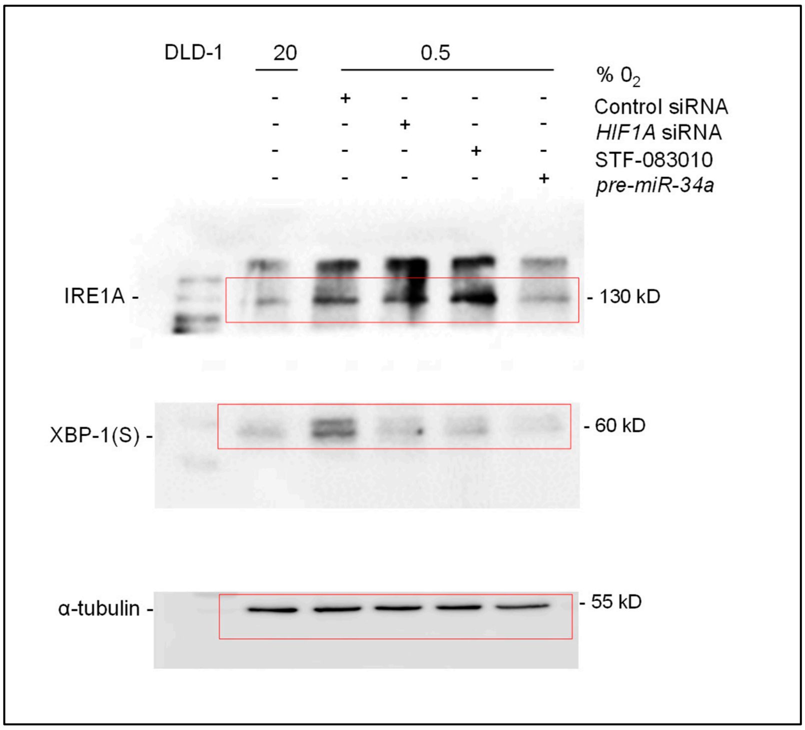

Related to Figure 7a

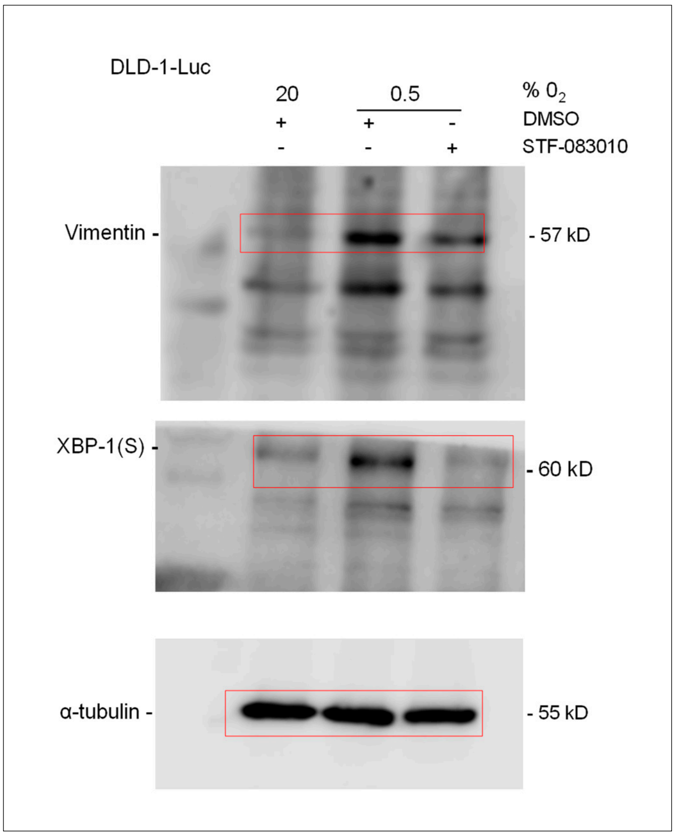

Related to Figure 8f

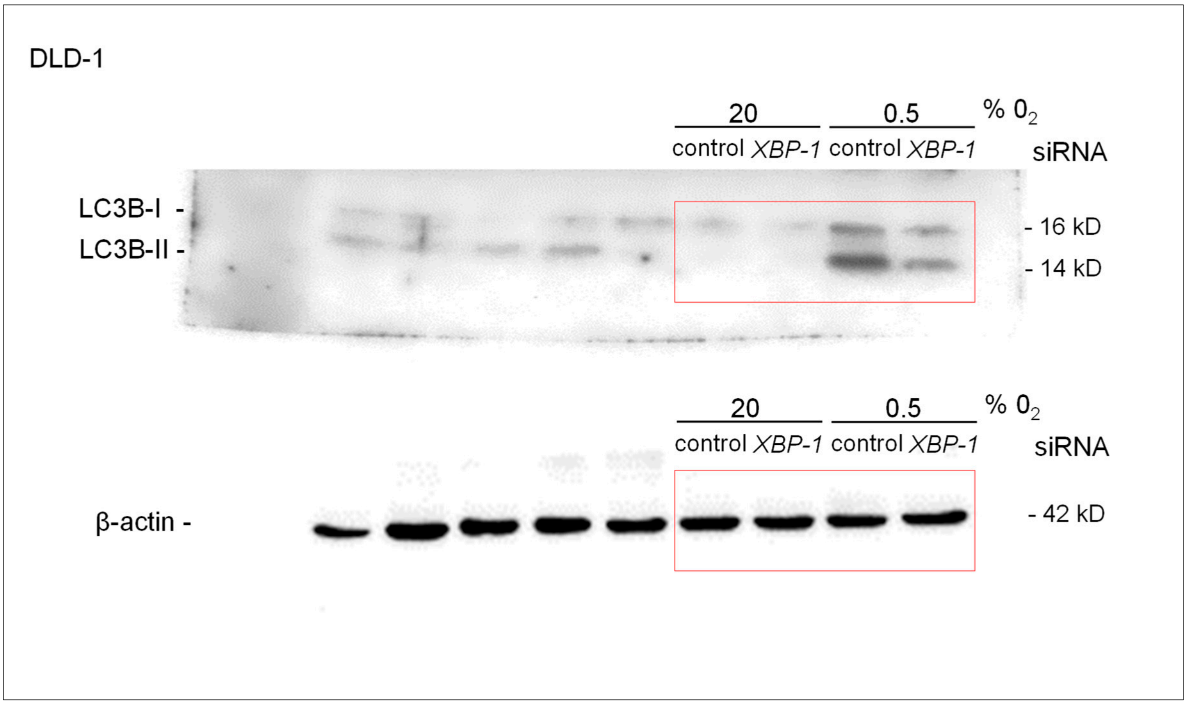

Related to Figure S1a

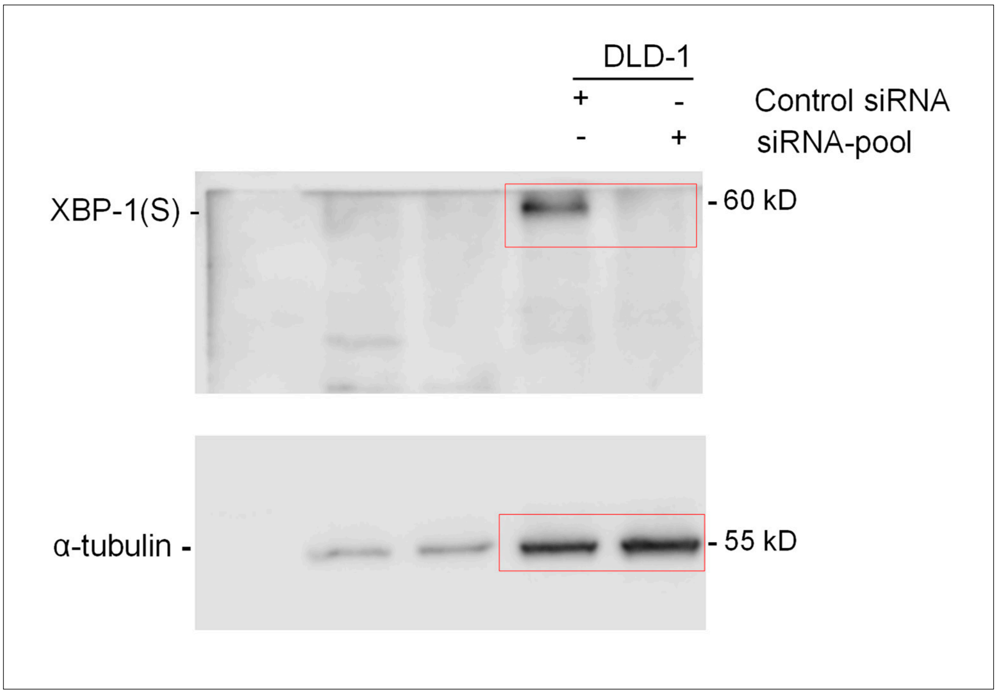

Related to Figure S1d

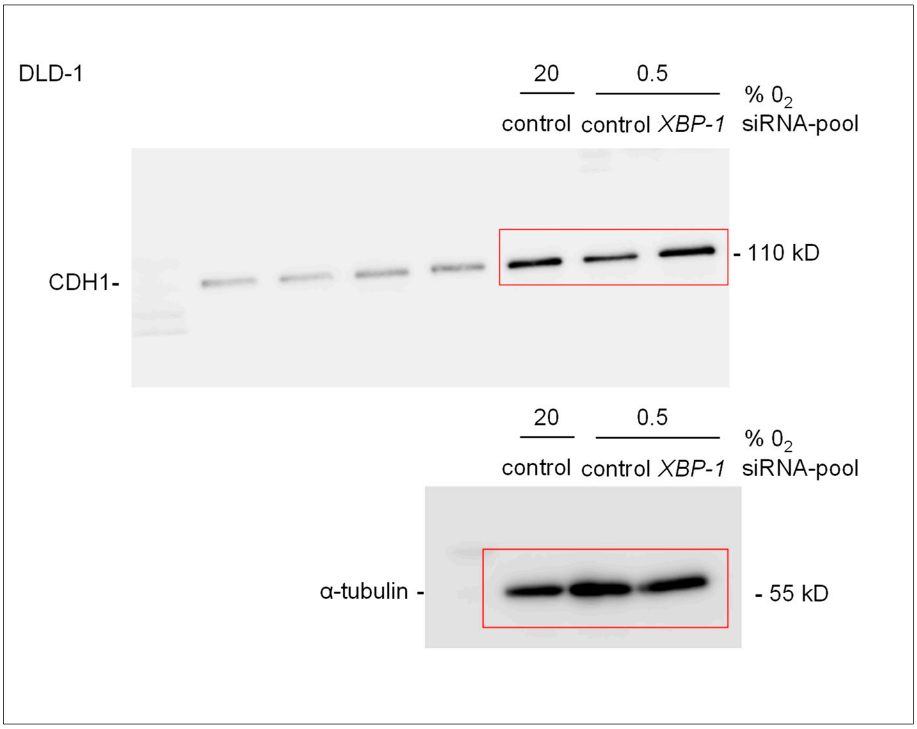

Related to Figure S2e

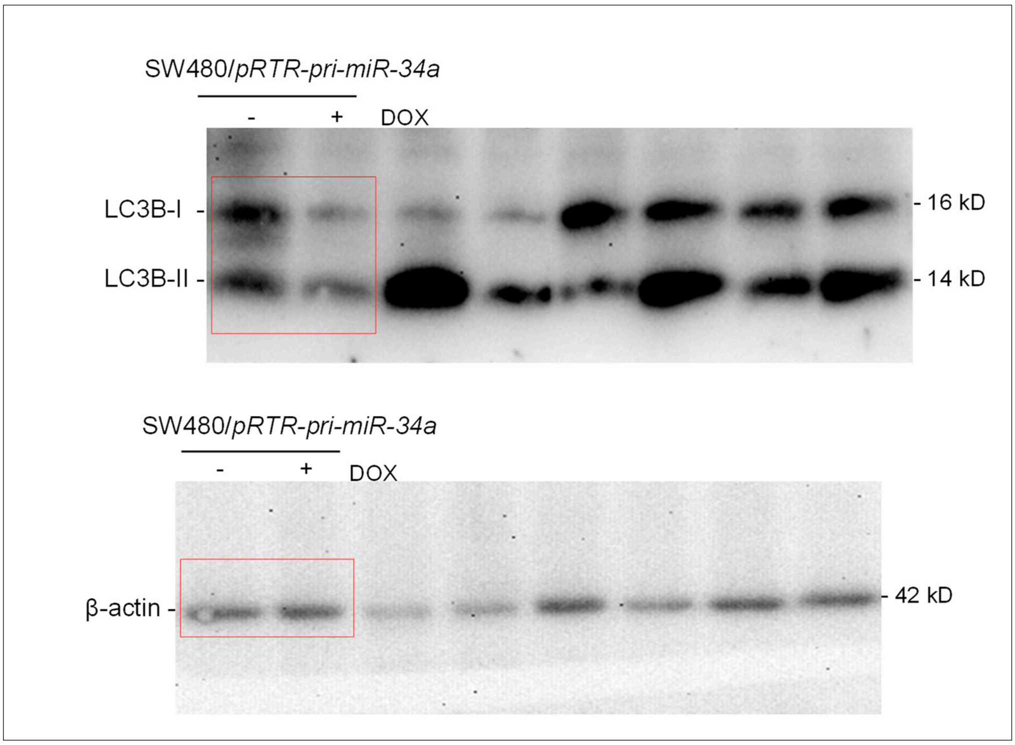

Related to Figure S2f

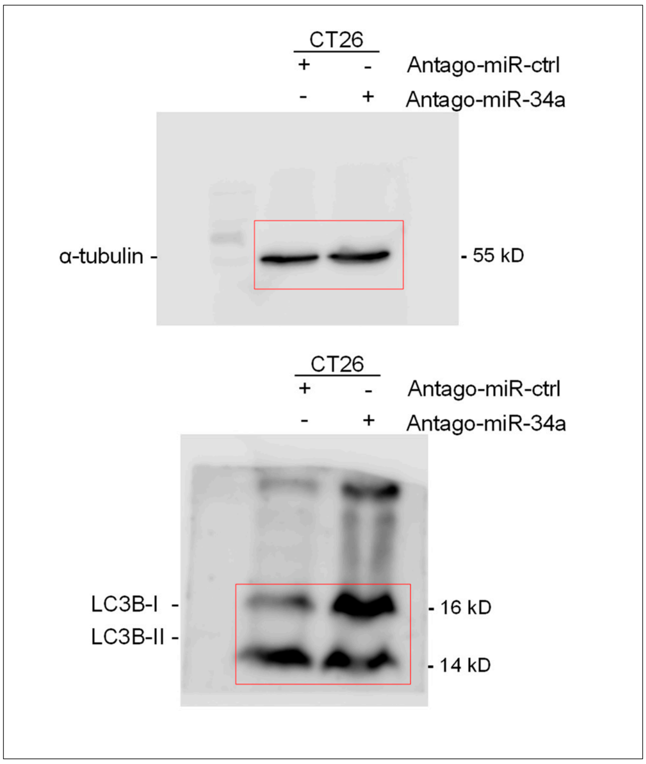

Supplementary Table S1: Oligonucleotides used for quantitative real-time PCR analyses

gene	forward (5' – 3')	reverse (5' – 3')
human *GAPDH*	GTTGCCATCAATGACCCCTT	CTCCACGACGTACTCAGCG
human *XBP-1*	AAGCCAAGGGGAATGAAGT	CCAGAATGCCCAACAGGATA
human *XBP1-(S)*	GCTGAGTCCGCAGCAGGTG	GCTGGCAGGCTCTGGGGAAG
human *IRE1A*	CGGGAGAACATCACTGTCCC	CCCGGTAGTGGTGCTTCTTA
human *pri-miR34a*	CGTCACCTCTTAGGCTTGGA	CATTGGTGTCGTTGTGCT
human *VIMENTIN*	TACAGGAAGCTGCTGGAAGG	ACCAGAGGGAGTGAATCCAG
human *SNAIL*	GCACATCCGAAGCCACAC	GGAGAAGGTCCGAGCACAC
human *SLUG*	GGGGAGAAGCCTTTTTCTTG	TCCTCATGTTTGTGCAGGAG
mouse *Xbp-1(S)*	CTGAGTCCGCAGCAGGT	TGTCAGAGTCCATGGGAAGA
mouse *Ire1a*	CCGAGCCATGAGAAACAAGAA	GGGAAGCGGGAAGTGAAGTAG
mouse *TUBULIN*	AGTAAACCGTAGCCATGAGG	CCTCCCAGAACTTAGCACC

Supplemental Table S2: List of antibodies

Primary antibodies

epitope	source	company	catalog no.	use	dilution mouse	dilution human
α-Tubulin	mouse	Sigma-Aldrich	#T9026	WB	1:1000	1:1000
B-actin	rabbit	Sigma Aldrich	# A2066	WB	1:1000	1:1000
E-cadherin	mouse	Invitrogen	# 334000	IF		1:50
XBP-1U (29 kda)	rabbit	Cell signaling	#12782	WB	1:1000	1:1000
XBP-1S(56 kda)	rabbit	Cell signaling	#12782	WB, IHC	1:1000, 1:500	1:1000
IRE1A	rabbit	Cell signaling	#3294	WB	1:1000	1:1000
IRE1A (pS724)	rabbit	abcam	# ab124945	WB		1:500
Vimentin	rabbit	Cell signaling	#5741	WB		1:1000
LC3B	rabbit	Cell signaling	#2775	WB, IF	1:1000, 1:500	1:1000

WB: Western Blotting; IHC: Immunohistochemistry; IF: Immunoflourescence

Secondary antibodies or conjugates

name	source	company	catalog no.	use	dilution
anti-mouse HRP	goat	Promega	#W4021	WB	1:10 000
anti-rabbit HRP	goat	Sigma Aldrich	#A0545	WB, ICH	1:1000
Alexa Flour 555 conjugated anti-rabbit	goat	Invitrogen	#A11034	IF	1:500
Alexa Flour 555 conjugated anti-mouse	goat	Invitrogen	#A21422	IF	1:500

WB: Western Blotting; IHC: Immunohistochemistry; IF: Immunoflourescence

Supplemental Table S3: Oligonucleotides used for qChIP

gene	forward (5' – 3')	reverse (5' – 3')
16q22	CTACTCACTTATCCATCCAGGCTAC	ATTTCACACACTCAGACATCACAG
UPRE	TCGCATCTTGTTGAATCCGG	AGGGCCTCTCGCCTGGA

Supplemental Table S4: Oligonucleotides used for cloning and mutagenesis of *XBP-1* and *IRE1A* 3'UTRs

gene	forward (5' – 3')	reverse (5' – 3')
Human *XBP-1 3'UTR*	GGACTAGTCCACATATATACCAAGCCCC	CGAGCTCCAGAAGCTACACTAGCAGG
Human *XBP-1 3'UTR mutant*	CTGCTTTCATCCAGCTCGCGAGCAAAGCCATCTTCCTG	CAGGAAGATGGCTTTGCTCGCGAGCTGGATGAAAGCAG
Human *IRE1A 3'UTR*	GGACTAGTCTCCTGGCAGGAAGTCATCAG	CGAGCTCCCTTTATTGTGGTTGGCTCGAC
Human *IRE1A 3'UTR mutant*	CCCTTTTCCTTGACTATTACACGTGGTGGAGGATAGCAGAG	CTCTGCTATCCTCCACCACGTGTAATAGTCAAGGAAAAGGG

Supplemental Table S5: Vectors used in this study

Name	Insert	Reference
pRTR	/	[40]
pRTR/*p53-VSV*	human *TP53*	[41]
pRTR/*pri-miR34a*	human *pri-miR-34a*	[42]
